# MT-100, a human Tie2-agonistic antibody, improves penile neurovasculature in diabetic mice via the novel target Srpx2

**DOI:** 10.1038/s12276-024-01373-1

**Published:** 2025-01-01

**Authors:** Fang-Yuan Liu, Young-Lai Cho, Fitri Rahma Fridayana, Lashkari Niloofar, Minh Nhat Vo, Yan Huang, Anita Limanjaya, Mi-Hye Kwon, Jiyeon Ock, Seon-Jin Lee, Guo Nan Yin, Nam-Kyung Lee, Ji-Kan Ryu

**Affiliations:** 1 National Research Center for Sexual Medicine and Department of Urology, Inha University College of Medicine, Incheon, Republic of Korea; 2 Environmental Disease Research Center, Korea Research Institute of Bioscience and Biotechnology, Daejeon, Republic of Korea; 3 Program in Biomedical Science & Engineering, Inha University, Incheon, Republic of Korea; 4 Department of Functional Genomics, University of Science and Technology, Daejeon, Republic of Korea; 5 Biotherapeutics Translational Research Center, Korea Research Institute of Bioscience and Biotechnology, Daejeon, Republic of Korea; 6 MabTics Co., Ltd., Daejeon, Republic of Korea

**Keywords:** Antibody therapy, Mechanisms of disease

## Abstract

Diabetes is an incurable, chronic disease that can lead to many complications, including angiopathy, peripheral neuropathy, and erectile dysfunction (ED). The angiopoietin-Tie2 signaling pathway plays a critical role in blood vessel development, formation, remodeling, and peripheral nerve regeneration. Therefore, strategies for activating the Tie2 signaling pathway have been developed as potential therapies for neurovascular diseases. Here, we developed a human Tie2-agonistic antibody (MT-100) that not only resists Ang-2 antagonism and activates Tie2 signaling but also regulates a novel target, sushi repeat-containing protein X-linked 2 (Srpx2). This regulation led to the survival of vascular and neuronal cells, a reduction in the production of reactive oxygen species (ROS), activation of the PI3K/AKT/eNOS signaling pathway, increased expression of neurotrophic factors, and ultimately alleviation of ED in diabetic mice. Our findings not only provide conclusive evidence that MT-100 is a promising therapeutic strategy for the treatment of diabetic ED but also suggest it has substantial clinical applications for other complications associated with diabetes.

## New Antibody Activates Tie2 to Combat Diabetic Complications

Erectile dysfunction is a condition where men have difficulty maintaining an erection. It is common among men with diabetes, often due to severe damage to blood vessels. Researchers found a need for better therapies for diabetic ED. They created a new humanized antibody, MT-100, which targets the Tie2 receptor. The Tie2 receptor is important for keeping blood vessels stable and protecting nerves. In their study, they tested MT-100 on diabetic mice. After injecting MT-100, the mice showed better erectile function due to improved blood vessel and nerve growth. MT-100 also helped cells survive and reduced oxidative stress, which is damage caused by harmful molecules. The study suggests MT-100 could be a promising treatment for diabetic ED by supporting blood vessel and nerve health. Future studies might look at MT-100 for other blood vessel diseases.

This summary was initially drafted using artificial intelligence, then revised and fact-checked by the author.

## Introduction

Erectile dysfunction (ED) is defined as the inability to achieve and/or maintain an erection sufficient for satisfactory sexual intercourse^1^. The global prevalence of ED is expected to reach 322 million cases by 2025^2^. As one of the major complications of diabetes mellitus (DM) in males, the incidence of ED among male diabetic patients is as high as 75%^3^. Although phosphodiesterase type 5 (PDE5) inhibitors are excellent first-line treatments, 50% of ED patients respond poorly because of severe vascular or nerve damage caused by DM, cardiovascular disease, and metabolic syndrome. Recent preclinical studies using angiogenic and neurotrophic factors, such as vascular endothelial growth factor (VEGF), cartilage oligomeric matrix protein (COMP)-angiopoietin-1, leucine rich alpha-2-glycoprotein 1, and brain-derived neurotrophic factor (BDNF), to treat ED have been somewhat successful^4–7^. However, owing to several limitations of protein therapies, such as low yield and purity in production, low stability, and the fact that recombinant proteins cannot effectively reach their targets because of poor pharmacokinetic (PK) profiles^8,9^, more effective treatment strategies, such as antibody therapies, are needed. Antibody therapeutics have become major contributors to the biopharmaceutical market in recent years because of their high safety profile, low toxicity, high refractive index, and single biological function^10^. However, studies of antibodies are all in their infancy, and few studies have investigated their application to treat ED^11–13^.

Angiopoietin (Angpt)-Tie receptor 2 (Tie2) signaling has been shown to promote vascular stability, vascular maturation, and endothelial cell survival^14^. Moreover, the neuroprotective and neurotrophic effects of this signaling pathway have only recently been discovered^15^. Tie2 is a receptor tyrosine kinase activated by higher-order oligomeric angiopoietin-1^16^. Most previous efforts to develop Tie2 agonists have focused on Angpt fibrinogen-like domain (FLD)-based protein engineering, such as COMP-angiopoietin-1^17^, cartilage matrix protein-angiopoietin-1^18^, Hepta-angiopoietin-1^19^, and FLD scaffold nanoparticles^20^. However, their clinical applications must overcome several limitations, such as the difficulties of large-scale production of native Ang1, a too-short half-life, excessive nonspecific binding to most tissues, and potential immunogenicity^14,17,21,22^. Therefore, agonistic antibodies are considered useful alternatives to natural ligands for receptors, as they can sustainably activate specific receptors, inducing downstream signaling pathways and cellular responses^14^. Furthermore, these antibodies are resistant to degradation in serum and have molecular characteristics that ensure long-term retention in circulation^14^.

More recently, Jo et al. developed a novel humanized Tie2-agonistic antibody (hTAAB) via mouse hybridoma technology and activity-based screening. It targets the membrane proximal fibronectin type III domain of Tie2, which is distinct from the Angpt-binding site, resulting in Tie2 activation^16^. Therefore, the development of Tie2-agonistic antibodies is a feasible way to advance research on the treatment of various vascular diseases for which vascular stabilization and normalization is therapeutically necessary, including ED. To the best of our knowledge, no Tie2-agonistic antibodies have been previously developed for the treatment of diabetic ED. Furthermore, the therapeutic mode of action of Tie2-agonistic antibodies in a diabetic ED model has been elusive.

Here, we developed a fully human Tie2-agonistic antibody, designated MT-100, that binds human Tie2 with cross-reactivity for various species of Tie2 and induces Tie2 phosphorylation to activate downstream signaling pathways. We aimed to evaluate the efficacy of MT-100 in promoting penile vascular neurogenesis to restore erectile function in a mouse model of diabetic ED.

First, we demonstrated that MT-100 can activate the Tie2 signaling pathway in human umbilical vein endothelial cells (HUVECs) and 3T3-J2 cells. Second, we demonstrated that two exogenous injections of MT-100 improved erectile function by enhancing neurovascular regeneration in cavernous tissue. RNA sequencing analysis revealed that these effects of MT-100 on penile neurovascular regeneration were sushi repeat-containing protein X-linked 2 (Srpx2)-dependent and that the expression of Srpx2 in the penis of diabetic mice is significantly lower than that in the penis of normal mice. Furthermore, exogenous injection of MT-100 promoted the expression of Srpx2, nerve growth factor (NGF), BDNF, and neurotrophin-3 (NT3), and activated the PI3K‒AKT signaling pathway to restore penile erectile function. These results provide important molecular and mechanistic insights into MT-100-mediated neurovascular regeneration in DM-induced ED.

## Materials and methods

### Ethics statement and study design

The health and behavior of all of the animals were monitored every day^7^, and all of the experiments performed in this study were approved by the Institutional Animal Care and Use Committee of our university (Assurance Number: 230209-858.). Eight-week-old male C57BL/6 J mice (20–25 g, Orient Bio, Inc., Seongnam, Korea) were used in this study. The mice were fed commercial standard laboratory food and water and maintained at room temperature (23 ± 2 °C) with 40–60% humidity, specific pathogen-free conditions, and 12-h light/dark cycles. The cages, bedding, food, and water were strictly disinfected. All animals were operated on under general anesthesia with intramuscular injection of ketamine (100 mg/kg, Yuhan Corp., Seoul, Korea) and xylazine (5 mg/kg, Bayer Korea, Seoul, Korea) prior to model preparation. Type 1 DM was induced by the intraperitoneal injection of multiple low doses of streptozotocin (STZ, 50 mg/kg body weight in 0.1 M citrate buffer, pH 4.5; Sigma‒Aldrich, St. Louis, MO, USA) for 5 consecutive days. Fasting and postprandial blood glucose levels were measured 8 weeks after STZ injection via an Accu-Check blood glucose meter (Roche Diagnostics, Mannheim, Germany). The mean systolic blood pressure (MSBP) was evaluated via a noninvasive tail-cuff system (Visitch System, Apex, NC, USA). To ensure the availability of diabetic mice, we examined fasting and postprandial blood glucose concentrations, which were significantly higher in STZ-induced diabetic mice than in age-matched nondiabetic controls. The body weights of diabetic mice were significantly lower than those of age-matched nondiabetic controls. However, these parameters did not change significantly, regardless of MT-100 treatment or lentivirus infection. There were no significant differences in the mean systolic blood pressure (MSBP) between the experimental groups **(**Supplementary Tables 1 and 2).

To test the ability of human Tie2-agonistic antibody (MT-100, provided by Mabtics Co., Ltd., South Korea, Daejeon) to alleviate erectile function in diabetes-induced ED mice, the STZ-induced type 1 diabetic mice received two intracavernous injections of PBS (Gibco, Carlsbad, CA, USA), NC (10 μg in 20 μL of PBS), or MT-100 (days −3 and 0; 1 μg, 10 μg, and 20 μg in 20 μL of PBS). For lentivirus infection, scrambled control shRNA (shCon) lentivirus particles (Santa Cruz Biotechnology, Dallas, TX, USA) or mouse lentivirus containing shRNA targeting Srpx2 (shSrpx2, OriGene Technologies Inc., Rockville, USA) were intracavernously injected at different transduction units (TU; 5 × 10^3^ TU/mL, 2.5 × 10^4^ TU/mL, 5 × 10^4^ TU/mL lentivirus). The sequence of the shSrpx2 construct used was ACCAGTCCACTGACTCAGAGAGGTGCTCT. All knockdown experiments were performed at least 3 days after lentivirus infection. We compressed the penis with a vascular clamp at the base immediately before injection and then used a 30-gauge insulin syringe for intracavernous injection. After the injection, the clamp remained in place for 30 min to restrict blood flow out of the penis. After 2 weeks, we evaluated erectile function in the mice via electrical stimulation of the cavernous nerve. All animals were euthanized by 100% CO_2_ gas replacement at 10–30% container volume/minute, and cessation of the heartbeat and respiratory arrest were confirmed before harvesting the tissues. All experiments in this study were performed in a blinded manner, and no mice died during the experiments.

### Production of recombinant proteins

The extracellular domain of human Tie2 (hTie2-ECD) was subcloned and inserted into the pFUSE-hIgG1 expression vector (InvivoGen, San Diego, CA, USA) to generate Fc-fused hTie2 (hTie2-ECD-Fc). The Fc region was replaced with a 6× His-tag, and hTie2-ECD was subcloned to generate hTie2 tagged with 6× His (hTie2-His). The vectors constructed to produce hTie2-ECD-Fc or hTie2-His were transfected into HEK293-F cells using Lipofectamine 2000 (Thermo Fisher Scientific, Fremont, CA, USA) according to the manufacturer’s instructions. The supernatants were collected, filter sterilized via 0.22-μm filters (Millipore, Temecula, CA, USA), and purified through an NGC Quest 100 column (Bio-Rad, Hercules, CA, USA) equipped with a MabSelect^TM^ PrismA column (Cytiva, St. Louis, MO, USA) for hTie2-ECD-Fc or a HisTrap^TM^ HP column (Cytiva) for hTie2-His. The purified proteins were dialyzed against PBS (pH 7.4) and analyzed via SDS‒PAGE.

### Selection and screening of Tie2-binding antibodies

For the selection of antibodies directed toward hTie2, a naive human scFv phage display library from pooled healthy donors was constructed via the pADL-22c phagemid vector (Antibody Design Labs, San Diego, CA, USA), and phage display panning was performed as described previously with some modifications^23^. Briefly, purified hTie2-ECD-Fc was prepared at 10 μg/mL in phosphate-buffered saline (PBS, pH 7.4) and an immunotube (Thermo Fisher Scientific) coated by overnight incubation at 4 °C. Phages rescued from the library were blocked in PBS and 2% nonfat milk (MPBS) for 1 h, followed by incubation within the hTie2-coated immunotube for 1 h. The immunotubes were washed with PBS supplemented with 0.05% Tween 20 (PBST), and the bound phages were eluted with 100 mM triethylamine and propagated as described previously^23^. To screen individual scFvs, single colonies were picked from the third-round output and inoculated into 150 μL of 2xYT/ampicillin/2% glucose in a 96-well U-bottom culture plate. After incubation for 3 h, the bacteria were pelleted via centrifugation, resuspended in 150 μL of 2xYT/ampicillin supplemented with 1 mM isopropyl β-D-1-thiogalactopyranoside (IPTG), and grown overnight at 30 °C with shaking and proper aeration. The plate was subsequently centrifuged, and scFvs were extracted from the bacterial pellets using 100 μL of periplasmic extraction buffer (PEB; 30 mM Tris-HCl/20% sucrose/1 mM EDTA, pH 8.0). The extracted scFvs were subjected to screening via ELISA with an HRP-conjugated anti-HA antibody (Sigma‒Aldrich). To assess the binding activity of the screened scFvs toward the extracellular domain of mouse Tie2 (mTie2-ECD), clones that demonstrated at least 5-fold binding over background were subjected to Sanger sequencing (Bioneer Inc., Daejeon, Korea) using a pelB-Forward primer (5′-AATACCTATTGCCTACGGCTG-3′) to identify the variable heavy (VH) or variable light (VL) region sequence.

### Transient expression and purification of IgGs

The VH and VL chains of each scFv antibody were amplified by PCR and subcloned and inserted into heavy chain (HC) and light chain (LC) expression vectors, respectively, as described previously^24^. For the expression of whole IgGs, a pair of HC and LC expression vectors was cotransfected into Expi293F cells (1 × 10^8^) and cultured for 5 days. The supernatant was centrifuged at 4000 rpm for 40 min and filtered through a 0.22-μm bottle-top vacuum filter. IgGs were purified using an NGC Quest 100 equipped with a MabSelect^TM^ PrismA column (Cytiva) and prepared by dialysis against PBS (pH 7.4).

### Cell culture and treatment

Primary MCECs were prepared and maintained as described previously^25^. Briefly, penis tissue was harvested, and then the glans, urethra, and dorsal neurovascular bundle were removed and the cavernous tissue was placed into a sterilized tube containing Hank’s balanced salt solution (HBSS; Gibco). The cavernous tissues were cut into 1- to 2-mm fragments, placed in 60-mm dishes, and covered with Matrigel (Becton Dickinson, Mountain View, CA, USA). Tissues were cultured with complement M199 medium (Gibco) containing 20% fetal bovine serum (FBS, Gibco), 0.5 mg/mL heparin (Sigma‒Aldrich), 5 ng/mL recombinant human vascular endothelial growth factor (VEGF, R&D Systems Inc., Minneapolis, MN, USA), and 1% penicillin/streptomycin (Gibco) in a 5% CO_2_ atmosphere at 37 °C. After the cells reached 80–90% confluence on the bottom of the 60-mm cell culture dishes (~2 weeks of culture), the sprouting cells were subcultured into other cell culture dishes coated with 0.2% gelatin (Sigma–Aldrich). Cells from passages 2 to 4 were available for all experiments.

HUVECs (Lonza, Cohasset, MN, USA) were cultured according to ATCC guidelines and were used between passages 2 and 7 for this study. Mouse Schwann cells (MSCs; ScienCell Research Laboratories, San Diego, CA, USA) were cultured according to the manufacturer’s guidelines and were used between passages 2 and 5 for this study. A mouse neuroblastoma line (N2a, ScienCell Research Laboratories) was cultured according to guidelines and was used between passages 2 and 5 for this study.

Primary MCPs were prepared and maintained as described previously^26^. Briefly, penis tissue was cut into several pieces of approximately 1 mm, and the tissue fragments were settled by gravity into a collagen I-coated 35-mm cell culture dish (BD Biosciences). After incubation with 300 µL of DMEM supplemented with 10% FBS, 1% penicillin/streptomycin, and 10 nM human pigment epithelium-derived factor (Sigma‒Aldrich) for 5 min at 37 °C, we added an additional 900 µL of the complement DMEM and incubated the cells at 37 °C in a 5% CO2 atmosphere. The culture medium was changed every 2 days. After the cells reached confluence and spread to the bottom of the dish, only the sprouting cells were subcultured. Sprouting cells were seeded into a dish coated with 50 µL/ml collagen I (Advanced BioMatrix). Cells between passages 2 and 4 were used for experiments.

To mimic DM-induced angiopathy, the serum-starved cells were exposed to normal glucose (NG, 5 mM glucose; Sigma‒Aldrich) or high glucose (HG, 30 mM glucose) for at least 72 h at 37 °C in a humidified 5% CO_2_ atmosphere^27^.

For the cell survival assay, HUVECs were analyzed as described previously with some modifications^28^. Briefly, HUVECs were plated at a density of 2 × 10^5^ cells/well in 24-well plates. The next day, the cells were serum deprived for 2 h in serum-free medium (M199) and then treated with MT-100 IgG at various concentrations for 40 h at 37 °C in a humidified 5% CO_2_ and 1% O_2_ atmosphere. The viability of the HUVECs was measured via crystal violet staining.

Tube formation assays were performed as described previously^29^. Briefly, 50 μL of growth factor-reduced Matrigel (Becton Dickinson) was dispensed into a 96-well plate at 4 °C. After gelling for at least 10 min at 37 °C, pretreated MCECs or HUVECs (exposed to NG or HG conditions with or without NC or MT-100 treatment) were seeded onto the gel at 5 × 10^4^ cells/well in 150 µL of M199 medium containing 2% FBS. Tube formation was monitored for 8–24 h, and images were taken with a phase-contrast microscope (CKX41, Olympus, Tokyo, Japan). The number of master junctions was counted in four separate experiments in a blinded manner using ImageJ software (National Institutes of Health [NIH] 1.34, http://rsbweb.nih.gov/ij/).

For the cell migration assay, the SPL ScarTMBlock system (SPL Life Sciences, Pocheon-si, Gyeonggi-do, Korea) was placed in 60 mm culture dishes. Pretreated MCECs or HUVECs (exposed to NG or HG conditions with or without NC or MT-100 treatment) were seeded into the block system at >90% confluence as described previously^30^. After 5 h, the blocks were removed, and the cells were further incubated in culture medium supplemented with 2% FBS and thymidine (2 mM, Sigma‒Aldrich) for 24 h. Images were taken with a phase-contrast microscope (Olympus), and the migrated cells were analyzed by measuring the ratio of cells that moved into the frame line for four separate block systems in a blinded manner using ImageJ software.

Cell apoptosis was assessed in mouse cavernous tissue, HUVECs, and MCECs via a terminal deoxynucleotidyl transferase-mediated dUTP nick-end labeling (TUNEL) assay kit (Chemicon, Temecula, CA, USA) according to the manufacturer’s instructions. Apoptotic cell digital images were observed at a screen magnification of 200× via a confocal fluorescence microscope (Nanoscope Systems, Inc.). The number of apoptotic cells from four separate experiments was determined in a blinded manner using ImageJ software.

To examine the effect of MT-100 on cavernous vascular permeability, HUVECs were cultured and exposed to NC (10 μg in 20 μL of PBS) or MT-100 (10 μg in 20 μL of PBS) under NG or HG conditions for at least 3 days. HUVECs were seeded at a density of 1 × 10^5^ cells/well on the bottom side of the Transwell insert (1.0 µm pore size; Becton Dickinson), which was placed in the 12-well plate overnight. The permeability was assayed by measuring the leakage of Evans blue (Sigma‒Aldrich) bound to bovine serum albumin (BSA, Bovogen Biologicals, VIC, Australia) as described previously^31^. The permeability was measured at selected time points.

For Tie2 knockdown, primary cultured MCECs and MSCs were transfected with siRNA against Tie2 (Thermo Fisher, San Jose, CA, USA) via Lipofectamine 2000 (Invitrogen, Carlsbad, CA, USA) according to the manufacturer’s instructions. Scrambled siRNA was used as a negative control. One day after transfection, the indicated groups were treated for another two days, after which the cells were harvested for Western blot analysis or other experiments.

For mouse Schwann cell neurite sprouting analysis, the MSCs were cultured in 60 mm cell culture dishes. One day after being transfected with scramble siRNA or Tie2 siRNA, the MSCs were treated with negative control IgG1 (NC), nerve growth factor-7S (NGF-7S, 10 ng/mL, R&D Systems Inc.), Ang1 (200 ng/mL, R&D Systems Inc.), or MT-100 (10 μg/mL) under normal glucose (NG, 5 mM) or high glucose (HG, 30 mM) conditions for another 3 days. At least three fields of isolated MSCs with clear boundaries were photographed using a phase-contrast microscope (CKX41, Olympus, Tokyo, Japan). The sprouting length of the MSCs was measured in a blinded manner via ImageJ software (National Institutes of Health [NIH] 1.34, http://rsbweb.nih.gov/ij/).

### Phospho-RTK array

A Proteome Profiler human phospho-receptor tyrosine kinase (RTK) array (R&D Systems) was used according to the manufacturer’s instructions. After HUVECs were treated with MT-100 IgG (5 µg/mL) for 30 min and lysed, the lysate containing 600 μg of protein lysate was incubated overnight with an antibody array containing 48 anti-RTK antibodies, 8 anti-phosphotyrosine positive control antibodies, and 5 negative control antibodies. The bound phospho-RTKs were detected with the anti-phospho-tyrosine antibody HRP. Blots were normalized to the eight antiphosphotyrosine antibody control spots per filter.

### Flow cytometry

Each full-length Tie2 cDNA from human, monkey, mouse, rat, dog, and pig was subcloned and inserted into the pcDNA3.4 vector. HEK-293T cells were transiently transfected with mock or Tie2-expressing vectors and further incubated for 24 h. The cells were collected using Accutase (Millipore), washed once with assay buffer (PBS containing 2% FBS), and resuspended in 100 µL of assay buffer for analysis. MT-100 IgG was incubated with the transfected cells for 30 min at 4 °C, and the cells were washed and labeled with an anti-human Fc antibody labeled with FITC (Thermo Fisher Scientific) for 30 min at 4 °C in the dark. After incubation, the cells were washed twice, resuspended in 400 µL of assay buffer, and analyzed using a NovoCyte 2000R combo (Agilent Technologies, Santa Clara, CA, USA). The data were analyzed and visualized using ACEA NovoExpress software (Agilent Technologies).

### Biolayer interferometry analysis

For affinity measurement of the antibodies, biolayer interferometry (BLI) assays were performed using a ForteBio Octet K2 system (ForteBio, Fremont, CA, USA) as described previously^32^. Briefly, recombinant human or mouse Tie2-Fc was immobilized on an amine-reactive second generation (AR2G) biosensor, and the ligand-loaded biosensor was allowed to bind to MT-100 IgG at various concentrations in PBS. The association and dissociation were monitored for 600 s each. The data were analyzed using ForteBio Data Analysis HT 12.0 software (ForteBio) to obtain the K_on_, K_off_, and K_D_ values.

### Immunoprecipitation

HUVECs and 3T3-J2 cells were incubated with or without various concentrations of MT-100 IgG or recombinant human angiopoietin-1 (Ang-1; R&D Systems) and then lysed with 600 µL of lysis buffer (50 mM Tris/HCl, pH 8.0; 1 mM ethylene diamine tetraacetic acid; 150 mM NaCl; 1 mM Na_3_VO_4_; 1 mM phenylmethylsulfonyl fluoride; and 1% NP-40). The cell lysates were centrifuged at 15,000 × *g* for 10 min, and the supernatants were subjected to immunoprecipitation at 4 °C overnight with an anti-Tie2 antibody (Becton Dickinson). After incubation, the antibody-Tie2 complex was collected via protein A Sepharose beads (Sigma‒Aldrich) at 4 °C for 4 h. The immunoprecipitates were then washed three times with lysis buffer, resuspended in SDS‒PAGE sample buffer containing β-mercaptoethanol, and further analyzed by Western blotting.

### Ex vivo study of the aortic ring assay and major pelvic ganglion (MPG) neurite sprouting assay

Aortic ring assays were performed as described previously^33^. Aortas harvested from 8-week-old C57BL/6 mice were cut into approximately 1-mm-thick rings, positioned in an SPLInsert™ standing confocal 6-well plate (SPL Life Sciences), and kept in place with an overlay of 10 µL of Matrigel. Aortic rings were cultured in complement M199 medium for 7 days under NG or HG conditions with or without NC and MT-100 treatment in a 5% CO_2_ atmosphere at 37 °C. The medium was changed every 2 days. Aortic segments and sprouting microvessels were then assessed via a phase-contrast microscope (Olympus). The results are expressed as areas of sprouting microvessels, which were measured from four separate experiments in a blinded manner using ImageJ software.

The mouse MPG tissues were prepared and maintained as described previously^34^. MPG tissues were harvested from 8-week-old C57BL/6 mice, cut into ~ 1-mm-thick rings, positioned in an SPLInsert™ standing confocal 6-well plate (SPL Life Sciences), and kept in place with an overlay of 10 µL of Matrigel for 5–10 min at 37 °C in a humidified 5% CO_2_ atmosphere. Next, 300 μL of complete neurobasal medium (Gibco) supplemented with 2% serum-free B-27 (Gibco), 0.5 nM GlutaMAX™-I (Gibco), and 1% penicillin/streptomycin (Gibco) was added. MPG tissues were treated with PBS (20 μL), NC (10 μg in 20 μL of PBS), or MT-100 (10 μg in 20 μL of PBS) under NG or HG conditions for 5 days in a 5% CO_2_ atmosphere at 37 °C. The medium was changed every 2 days. Neurite outgrowth segments were fixed in 4% paraformaldehyde for at least 30 min and immunostained with antibodies against neurofilaments (anti-NF, 1:50, Sigma‒Aldrich).

### Measurement of erectile function

Mouse erectile function was evaluated via the Biopac Student Lab System (Biopac Systems Inc., Goleta, CA, USA) as described previously^7^. The parameters were as follows: voltage, 5 V; frequency, 12 Hz; pulse width, 1 ms; and duration, 1 min. Mice were anesthetized with xylazine (5 mg/kg) and ketamine (100 mg/kg). The cavernous nerve was dissected and exposed, and then a bipolar platinum wire electrode was placed around the cavernous nerve and stimulated at the set value. Each electronic stimulus was repeated twice at approximately 10 min intervals. The maximum intracavernous pressure (ICP) and total ICP (area under the curve) were recorded. Systemic blood pressure variations were adjusted by the ratio of the maximal ICP or total ICP to the MSBP.

### Tracing FITC-labeled MT-100 in corpus cavernous tissues

The MT-100 and FITC conjugation mixture was prepared in advance according to the instructions of the FITC Conjugation Kit (Fast) - Lightning-Link® (Abcam, Cambridge, MA, USA). The tissues were harvested 0, 1, 6, and 12 h after intracavernous injection of the antibody mixture into normal mice and fixed in 4% paraformaldehyde overnight in the dark. Frozen 12-µm-thick sections were prepared. The slides were mounted in a solution containing 4,6-diamidino-2-phenylindole (DAPI, Vector Laboratories) for nuclear staining. Digital images were obtained via a confocal fluorescence microscope (Nanoscope Systems, Inc.).

### RNA sequencing assay and data analysis

RNA sequencing was performed as a custom service by E-Biogen, Inc. (Ebiogen, Inc., Seoul, Republic of Korea), as described previously^35^. The cavernosum tissues from control nondiabetic mice and mice with STZ-induced diabetes received two intracavernous injections of NC (10 μg in 20 μL of PBS) or MT-100 (days −3 and 0; 10 μg in 20 μL of PBS). Briefly, total RNA was isolated via TRIzol reagent (Invitrogen, Carlsbad, CA, USA). The RNA quality was assessed using an Agilent 2100 Bioanalyzer (Agilent Technologies), and the RNA was quantified using an ND-2000 Spectrophotometer (Thermo, Inc., Wilmington, DE, USA).

Libraries were prepared from total RNA using a SMARTer Stranded RNA-Seq Kit (Clontech Laboratories, Inc., Mountain View, CA, USA). mRNA was isolated using a Poly (A) RNA Selection Kit (Lexogen, Inc., Vienna, Austria), indexed with Illumina indices 1-12, and enriched via PCR. Libraries were assessed for mean fragment size using an Agilent 2100 Bioanalyzer (DNA High Sensitivity Kit) and quantified using a StepOne Real-Time PCR System (Life Technologies, Inc., Carlsbad, CA, USA). High-throughput sequencing was performed as paired-end 100 sequencing with a HiSeq 2500 system (Illumina, Inc., San Diego, CA, USA). FastQC (https://www.bioinformatics.babraham.ac.uk/projects/fastqc/) was used for quality control of the raw sequencing data. Adapters and low-quality reads (<Q20) were removed using FASTX_Trimmer (http://hannonlab.cshl.edu/fastx_toolkit/) and BBMap (https://sourceforge.net/projects/bbmap/), and trimmed reads were mapped to the reference with TopHat^36^. Gene expression levels were estimated on the basis of the read count (RC) and fragments per kilobase per million reads (FPKM), which were determined using BEDTools^37^ and Cufflinks^38^ and then normalized with EdgeR within R (https://www.r-project.org) quantile normalization. Data mining and graphic visualization were achieved with ExDEGA (E-Biogen, Inc., Seoul, Republic of Korea). The RNA sequencing data were stored in the Gene Expression Omnibus database (www.ncbi.nlm.nih.gov/geo accession no. GSE253248).

### Histological examination

For fluorescence examinations, mouse penile tissues were harvested and fixed in 4% paraformaldehyde overnight at 4 °C, and cell samples were fixed in 4% paraformaldehyde for 15 min at room temperature. The samples were blocked with 1% BSA (Sigma‒Aldrich) for 1 h at room temperature and incubated with primary antibodies against VE-cadherin (1:50, Santa Cruz Biotechnology), CD31 (1:50, Millipore), NG2 (1:50, Millipore), NF (1:50, Sigma‒Aldrich), neuronal nitric oxide synthase (nNOS, 1:50, Santa Cruz Biotechnology), phosphohistone H3 (PH3, 1:50, Millipore), claudin-5 (1:100, Invitrogen), occludin (1:100, Novus Biologicals, Centennial, Colorado, USA), nitrotyrosine (1:50, Millipore), or p-eNOS Ser^1177^ (1:500, Cell Signaling, Beverly, MA, USA) at 4 °C overnight as described previously^33^. After several washes with PBS (Gibco), the samples were incubated with fluorescein (FITC)-conjugated goat anti-Armenian hamster IgG (1:200, Jackson ImmunoResearch Laboratories, West Grove, PA, USA), donkey anti-rabbit Dylight 550 (1:200, Abcam), rhodamine (TRITC)-conjugated AffiniPure rabbit anti-mouse IgG (1:200, Jackson ImmunoResearch Laboratories), or donkey anti-mouse Alexa Fluor 488 (1:200, Jackson ImmunoResearch Laboratories) secondary antibodies for 2 h at room temperature. After several washes with PBS (Gibco), the samples were mounted in a solution containing DAPI (Vector Laboratories) for nuclear staining. The signals were visualized using a confocal microscope (Nanoscope Systems, Inc.). The samples were quantified in four separate experiments in a blinded manner using ImageJ software (National Institutes of Health [NIH] 1.34, http://rsbweb.nih.gov/ij/).

### In situ detection of superoxide anion

For superoxide anion detection, we used hydroethidine (Molecular Probes, Eugene, OR, USA), an oxidative fluorescent dye, to detect intracellular superoxide anions in situ as described previously^39^. Briefly, after the samples were stained with nitrotyrosine (a peroxynitrite marker, 1:50; Millipore), they were incubated with primary antibody and the corresponding secondary antibody. Before mounting, the samples were incubated with hydroethidine (1:10,000; Molecular Probes) for 30 min at room temperature in the dark. Finally, the tissue sections were mounted in a solution containing DAPI (Vector Laboratories) for nuclear staining. The fluorescent signals were visualized using a confocal microscope (Nanoscope Systems, Inc.). The expression of ethidium bromide was determined in four separate experiments in a blinded manner using ImageJ software (National Institutes of Health [NIH] 1.34, http://rsbweb.nih.gov/ij/).

### Western blot

For the immunoblot analyses, equal amounts of protein (50 µg/lane) were electrophoresed on sodium dodecyl sulfate‒polyacrylamide gels (4% to 20%) and transferred to polyvinylidene fluoride membranes. After blocking with 5% nonfat dry milk at room temperature for 1 h, the membranes were probed with the following primary antibodies: anti-p-Tie2 (1:500, R&D), anti-Tie2 (1:500, R&D), anti-p-eNOS Ser^1177^ (1:1000, Cell Signaling), anti-eNOS (1:1000, BD, Mountain View, CA, USA), anti-p-PI3KT^yr199^ (1:1000, Cell Signaling), anti-p-AKT^Ser473^ (1:1000, Cell Signaling), anti-AKT (1:1000, Cell Signaling), anti-p-ERK^Thr202/Tyr204^ (1:1000, Cell Signaling), anti-ERK (1:1000, Cell Signaling), anti-ADAM8 (1:1000, Invitrogen), anti-Hmox1 (1:500, Cell Signaling), anti-Srpx2 (1:1000, Abcam), anti-neurotrophin-3 (NT-3, 1:500; Santa Cruz Biotechnology), anti-brain-derived neurotrophic factor (BDNF, 1:500; Santa Cruz Biotechnology), anti-nerve growth factor (NGF, 1:500, Santa Cruz Biotechnology), and anti-β-actin (1:2000, Santa Cruz Biotechnology). The signals were visualized via an enhanced chemiluminescence (ECL) (Amersham Pharmacia Biotech, Piscataway, NJ, USA) detection system. The results were quantified by densitometry from four separate experiments and counted in a blinded manner using ImageJ software (National Institutes of Health [NIH] 1.34, http://rsbweb.nih.gov/ij/).

### Statistical analysis

The mean and SEM were obtained from at least four independent experiments. Unpaired *t* tests were used to compare two groups, and one-way ANOVA followed by Tukey’s post hoc test was used to compare more than two groups. The analysis was conducted via GraphPad Prism version 8 (GraphPad Software Inc., San Diego, CA, USA). *P* < 0.05 indicated statistical significance.

## Results

### Generation of a cross-species Tie2-agonistic antibody that inhibits vascular permeability and enhances endothelial cell survival

**Fig. 1:**
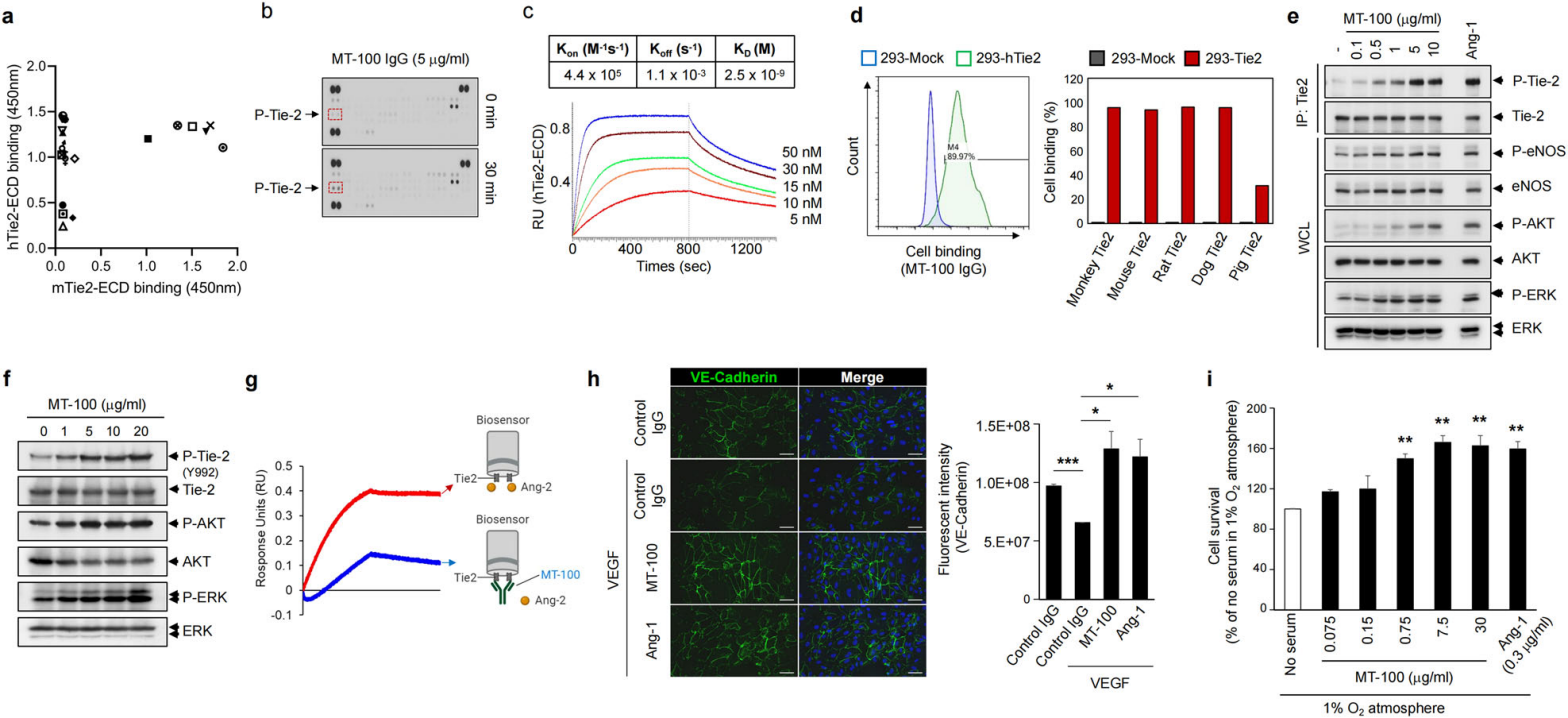
Generation and characterization of a cross-species Tie2-agonistic antibody. **a** Screening of Tie2-binding scFv antibodies. Three rounds of phage display panning were performed with hTie2-ECD-Fc, and individual scFvs were screened from the third-round output. The cross-species binding activity of the screened scFvs was evaluated using hTie2-ECD-Fc or mTie2-ECD-Fc via ELISA. **b** Phospho-RTK arrays in HUVECs. MT-100 was produced as an IgG and incubated with HUVECs. Cell lysates obtained before or after MT-100 treatment were used for RTK arrays. **c** Affinity measurement via BLI. Recombinant hTie2-ECD was immobilized on a biosensor tip, and the indicated concentrations of MT-100 were allowed to bind to the tip. The K_D_ (K_off_/K_on_) value was calculated via global fitting analysis. **d** Assessment of cross-species binding of MT-100. The full-length Tie2 of each species was transiently expressed in HEK-293T cells, and the binding of MT-100 was analyzed by flow cytometry. **e** Evaluation of Tie2 and cascade signaling activation in HUVECs. The indicated concentrations of MT-100 were incubated with HUVECs, and phosphorylated Tie2 was detected in the lysates of the cells via immunoprecipitation with an anti-Tie2 antibody. Phosphorylated eNOS, AKT, and ERK were detected in whole-cell lysates (WCLs). **f** Evaluation of Tie2 and cascade signaling activation in a mouse cell line. 3T3-J2 cells were treated with various concentrations of MT-100, and phosphorylated Tie2, AKT, and ERK were detected in WCLs. **g** Evaluation of the ability of MT-100 to block the Ang-2 and Tie2 interaction. Recombinant hTie2-ECD was immobilized on a biosensor, and buffer (red) or MT-100 (blue) was incubated with the biosensor. Ang-2 was further allowed to bind to each biosensor, and the binding kinetics were analyzed via biolayer interferometry. Illustrations were created with BioRender.com. **h** Assessment of endothelial cell integrity. HUVECs cultured under confluent conditions were incubated with the indicated treatments. Tight junction integrity was validated by VE-cadherin staining. Nuclei were stained with DAPI (blue). Scale bars, 100 µm. **i** Effect of MT-100 on endothelial cell survival. The indicated concentrations of MT-100 were incubated with HUVECs under hypoxic and serum-free conditions. Ang-1 was used as a positive control. Cell survival was analyzed via trypan blue staining. The data are presented as mean ± SEM (*n* = 3). The relative ratio in the control was set to 100. **P < 0.01. DAPI, 4,6-diamidino-2-phenylindole; HUVECs, human umbilical vein endothelial cells.

To identify novel cross-species Tie2-binding antibodies, we generated a recombinant extracellular domain of hTie2 fused with mouse Fc (hTie2-ECD-Fc) and performed phage display panning using a naive human single chain variable fragment (scFv) phage display library. Twenty-two hTie2-binding scFvs were screened from the selection outputs via ELISA, and six clones showed high binding activity toward the extracellular domain of mouse Tie2 (mTie2-ECD) (Fig. 1a). The Tie2-agonistic activity of the antibodies was assessed on the basis of phospho-RTK arrays using antibodies reformatted as an IgG. We found that an antibody named MT-100 could specifically induce the phosphorylation of human Tie2, with no phosphorylation activity toward other RTKs in the array (Fig. 1b). We evaluated the kinetics of the binding of MT-100 to hTie2-ECD via biolayer interferometry. Measurement of the interaction (K_on_) and dissociation (K_off_) revealed a value of 2.5 nM KD for MT-100 toward hTie2 (Fig. 1c). To assess the cross-species binding of MT-100, flow cytometry was performed using HEK-293 cells transiently expressing various species of Tie2. As shown in Fig. 1d, MT-100 exhibited a broad range of high binding activity to monkey, mouse, rat, and dog Tie2, as did hTie2, although it exhibited less binding to pig Tie2.

As Ang1/Tie2 signaling triggers the activation of ERK, AKT, and eNOS, we investigated Tie2 and the cascade signaling activation by MT-100. MT-100 treatment clearly induced the phosphorylation of not only Tie2 but also ERK, AKT, and eNOS in a dose-dependent manner, indicating that MT-100 acts in the same manner as Ang1 does (Fig. 1e). These characteristics were similar to those of mouse 3T3-J2 cells expressing mouse Tie2 (Fig. 1f).

Next, we performed biolayer interferometry to evaluate whether MT-100 interferes with the interaction between Tie2 and the antagonistic ligand angiopoietin-2 (Ang-2). Ang-2 alone or MT-100 plus Ang-2 was allowed to bind to hTie2 immobilized on a biosensor, and we found that MT-100 prebound to hTie2 could block the access of Ang-2 to hTie2 (Fig. 1g). In addition, tight junction maintenance by Tie2 activation was validated by immunocytochemical staining of VE-cadherin. In HUVECs subjected to hyperpermeable conditions induced by VEGF or Ang-1 (positive control), MT-100 treatment restored the expression of VE-cadherin, indicating that MT-100 can ensure the integrity of endothelial junctions (Fig. 1h). Furthermore, because MT-100 activates the AKT and eNOS signaling pathways, the survival of HUVECs was increased by incubation with MT-100 even under hypoxic and serum-free conditions (Fig. 1i). These results indicate that, as a Tie2 agonist, MT-100 can potently activate Tie2 and cascade signaling pathways to maintain endothelial permeability and increase cell survival.

### MT-100 induces angiogenesis and migration under high-glucose conditions in human and mouse endothelial cells

**Fig. 2:**
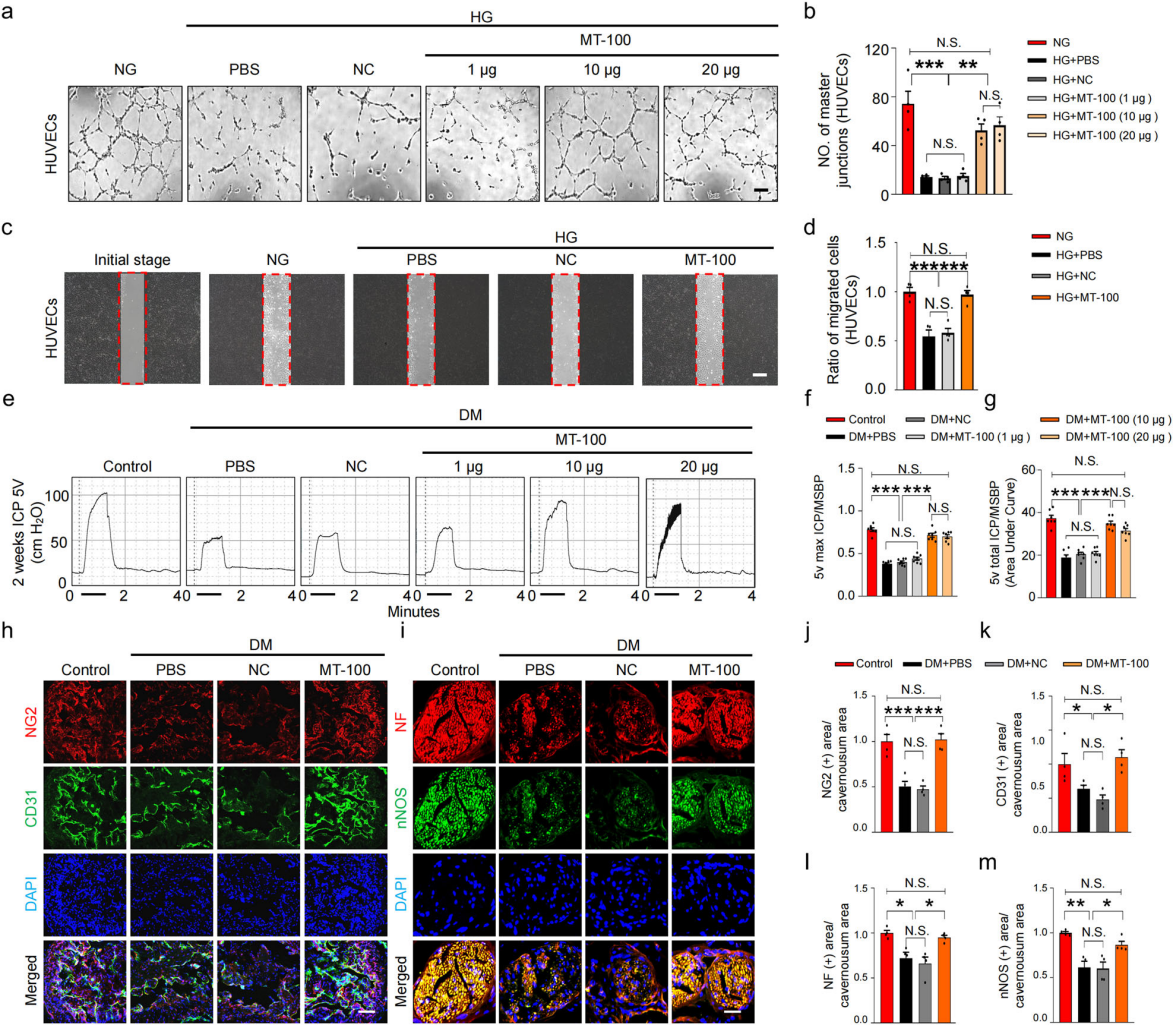
MT-100 induces neurovascular regeneration and improves erectile function under diabetic conditions. **a** Tube formation assay. HUVECs were treated with phosphate-buffered saline (PBS), negative control IgG1 (NC, 10 μg/mL), or MT-100 (1 μg, 10 μg, or 20 μg/mL) under normal glucose (NG) or high glucose (HG) conditions for 3 days. Scale bars, 100 µm. **b** Quantification of the number of master junctions per field (*n* = 4). **c** Migration of HUVECs 24 h after treatment with PBS, NC (10 μg/mL), or MT-100 (10 μg/mL) under NG or HG conditions for 3 days. Scale bars, 100 µm. **d** The ratio of migrated HUVECs in the frame line was quantified (*n* = 4). **e** Representative intracavernous pressure (ICP) responses of age-matched control and STZ-induced diabetic (DM) mice stimulated at 2 weeks after repeated intracavernous injection (days −3 and 0) of PBS (20 μL), NC (10 μg in 20 μL of PBS), or MT-100 (1 μg, 10 μg, or 20 μg in 20 μL of PBS) on days 0 and 3. The cavernous nerve was stimulated at 5 V. The stimulus interval is indicated by a solid bar. **f**, **g** Ratios of the mean maximal ICP (**f**) and total ICP (**g**, area under the curve) to mean systolic blood pressure were calculated for each group (*n* = 7). **h**, **i** Representative images of immunofluorescence staining of corpus cavernosum (**h**) and dorsal nerve bundle (**i**) tissues from age-matched control and DM mice 2 weeks after repeated intracavernous injection (days −3 and 0) of PBS (20 μL), NC (10 μg in 20 μL of PBS), or MT-100 (10 μg in 20 μL of PBS) for NG2 (red), CD31 (green), NF (red), or nNOS (green) after ICP studies. Nuclei were stained with DAPI (blue). Scale bars, 100 µm (**h**) and 25 µm (**i**). **j**‒**m** Quantitative analysis of corpus cavernosum pericyte (NG2), endothelial cell (CD31), and dorsal nerve bundle neuronal cell (NF and nNOS) content using ImageJ software. The data are presented as mean ± SEM (*n* = 4). The relative ratio in the control or NG groups was arbitrarily set to 1. *P < 0.05; **P < 0.01; ***P < 0.001. DAPI 4,6-diamidino-2-phenylindole, HUVECs human umbilical vein endothelial cells, N.S. not significant.

To determine whether the angiogenic effects of the human MT-100 antibody can act on both human and mouse cells, we treated HUVECs and MCECs with different doses of MT-100 under high-glucose (HG) conditions. Under HG conditions, the tube formation of HUVECs and MCECs was significantly reduced, whereas the angiogenic activity of HUVECs and MCECs was significantly increased after MT-100 treatment, especially at 10 μg/mL (Fig. 2a, b and Supplementary Fig. 1a, d). Furthermore, under HG conditions, MT-100 (10 μg/mL) promoted the migration of HUVECs and MCECs (Fig. 2c, d and Supplementary Fig. 1b, e). Consistent with the in vitro tube formation assay results, microvessel sprouting from aortic rings was severely reduced under HG conditions treated with PBS or negative control IgG1 (NC), and MT-100 treatment inhibited this reduction under HG conditions (Supplementary Fig. 1c, f). These findings provide evidence for the utility of MT-100 in the treatment of diabetic ED.

### Metabolic variables

Fasting and postprandial blood glucose concentrations were significantly greater in STZ-induced diabetic mice than in age-matched nondiabetic controls. The body weights of the diabetic mice were significantly lower than those of age-matched nondiabetic controls. However, these parameters did not change significantly, regardless of MT-100 treatment or lentivirus infection. There were no significant differences in the mean systolic blood pressure (MSBP) between the experimental groups (Supplementary Tables 1 and 2).

### MT-100 improves erectile function through neurovascular regeneration in STZ-induced diabetic mice

To determine the effect of MT-100 on the recovery of erectile function, we observed the distribution of exogenously injected MT-100 in the penis by intracavernously injecting the MT-100-FITC conjugation mixture and harvesting tissues at 0, 1, 6, and 12 h. We found that MT-100 mostly accumulated in the cavernous tissue and dorsal nerve bundle at 1 h and was almost completely absorbed at 12 h (Supplementary Fig. 2). Next, we evaluated erectile function during in vivo electrical stimulation of the cavernous nerve (5 V, 12 Hz, and 1 m/s) 2 weeks after intracavernous injection of PBS NC (10 μg in 20 μL of PBS) or MT-100 (days −3 and 0; 1 μg, 10 μg, and 20 μg in 20 μL of PBS) in age-matched control and diabetic mice (Fig. 2e–g). The ratios of the maximum ICP (Fig. 2f) and total ICP (Fig. 2g) to the mean systolic blood pressure (MSBP) were significantly lower in the diabetic group injected with PBS or NC than in the age-matched control group. These erectile parameters were significantly improved in the 10 µg and 20 µg MT-100 injection groups, reaching 90% of the control values in both groups. No significant recovery of erectile function was found in the 1 µg MT-100 injection group. Furthermore, we were unable to obtain functional results in the high-dose (50 μg) MT-100 injection group because of severe inflammation of the corpus cavernosum tissue, whereas the 20 µg MT-100 injection group also exhibited a certain inflammatory response. Therefore, we selected 10 µg of MT-100 as the optimal concentration for in vivo experiments.

Immunofluorescence staining of NG2 (pericyte marker), CD31 (endothelial cell marker), neurofilament-2000 (nerve fiber marker), and nNOS (nerve fiber marker) in cavernosum tissue revealed that MT-100 injection (10 μg) significantly restored pericyte, endothelial cell, and neuronal cell contents in diabetic mice (Fig. 2h–m). We further evaluated the effect of MT-100 on neurite growth in isolated major pelvic ganglion (MPG) explants exposed to HG conditions. Immunofluorescence staining of MPG tissue for NFs revealed that neurite sprouting was significantly reduced under HG conditions, whereas MT-100 treatment significantly induced neurite outgrowth under these conditions (Supplementary Fig. 3). In addition, we found that MT-100 induced the expression of the Tie2 receptor in the cavernosum and dorsal nerve bundles (Supplementary Fig. 4), which may further support the promotion of the Tie2 signaling pathway. To further determine whether MT-100 truly requires Tie2 receptors to promote vascular nerve regeneration, we transfected Tie2 siRNA into MCECs and MSCs to knockdown the expression of Tie2 receptors and then treated them with MT-100. We found that under Tie2 receptor knockdown conditions, the tube formation of MCECs and the nerve neurite sprouting of MSCs were significantly reduced (Supplementary Fig. 5). Taken together, these data indicate that MT-100 enhances neurovascular regeneration in a Tie2 receptor-dependent manner under high-glucose conditions and can restore erectile function by promoting vascularity and nerve regeneration in the penises of diabetic mice.

### MT-100 promotes cell survival by increasing proliferation and reducing apoptosis under diabetic conditions

**Fig. 3:**
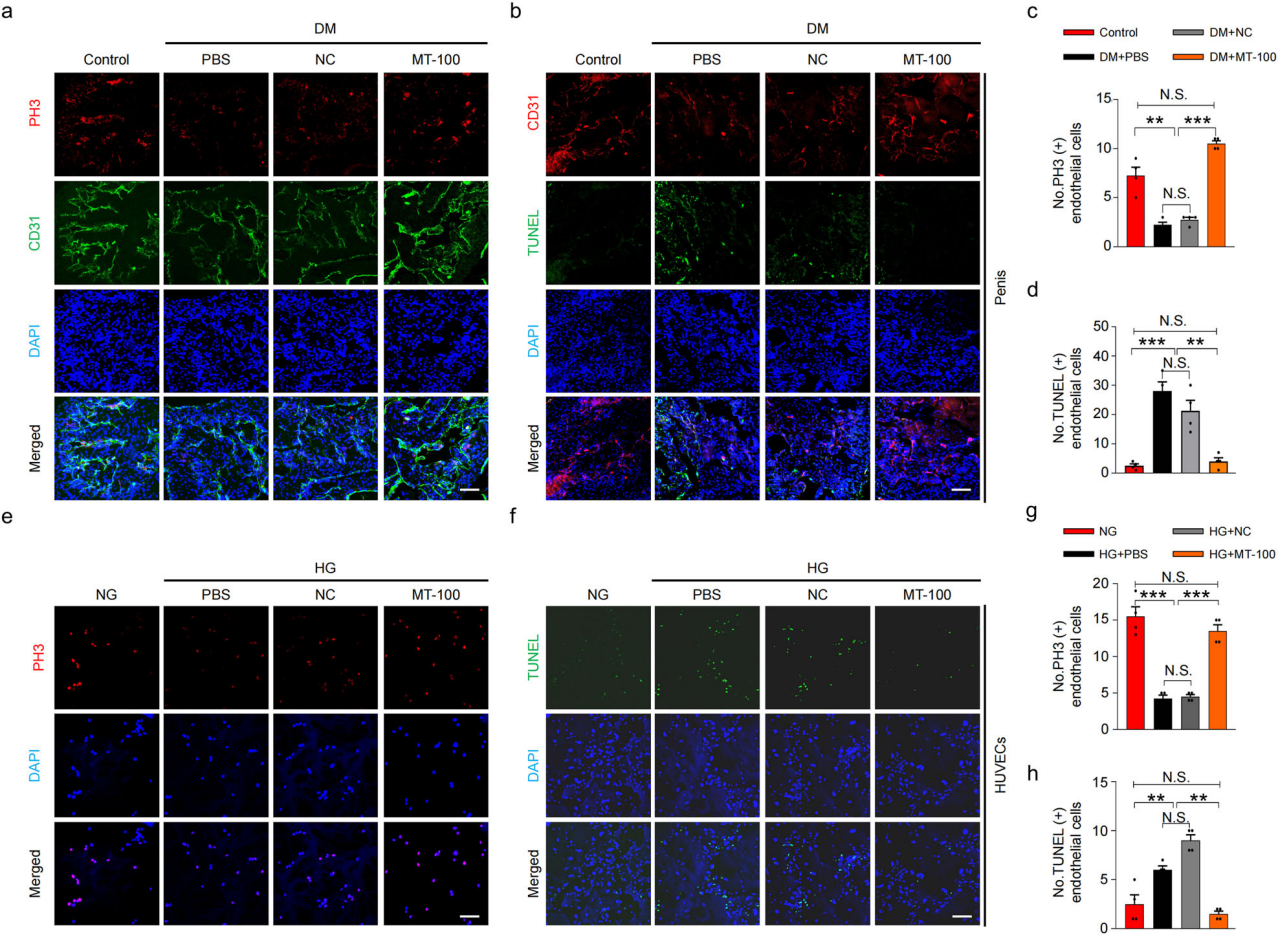
MT-100 increases proliferation and decreases apoptosis of corpus cavernosum endothelial cells under diabetic conditions. **a** Representative images of immunofluorescently stained corpus cavernosum tissues from age-matched control and STZ-induced diabetic (DM) mice 2 weeks after repeated intracavernous injection (days −3 and 0) of phosphate-buffered saline (PBS, 20 μL), negative control IgG1 (NC, 10 μg in 20 μL of PBS), and MT-100 (10 μg in 20 μL of PBS) for phospho-histone H3 (PH3; red) and CD31 (green) after ICP studies. **b** TUNEL assay (green) and CD31 (red) immunofluorescence staining under the same conditions described above. Nuclei were stained with DAPI (blue). Scale bars, 100 µm. **c**, **d** Number of PH3-positive (**c**) and TUNEL-positive (**d**) endothelial cells quantified by ImageJ software. The results are presented as mean ± SEM (*n* = 4). **e–h** In vitro studies of the proliferation assay (PH3) (**e**) and TUNEL assay (**f**) in HUVECs treated with PBS, NC (10 μg/mL), or MT-100 (10 μg/mL) under normal glucose (NG) or high glucose (HG) conditions for 3 days. Nuclei were stained with DAPI (blue). Scale bars, 25 µm. **g**, **h** Number of PH3-positive (**g**) and TUNEL-positive (**h**) HUVECs quantified via ImageJ software. The results are presented as mean ± SEM (*n* = 4). **P < 0.01; ***P < 0.001. DAPI 4,6-diamidino-2-phenylindole, TUNEL terminal deoxynucleotidyl transferase-mediated deoxyuridine triphosphate nick end labeling, HUVECs human umbilical vein endothelial cells, N.S. not significant.

The reduction in vascular and neuronal cells in DM is related to reduced proliferation and increased apoptosis^40^. To explore how MT-100 restores vascular and neuronal cell contents in diabetic mice, we examined cavernous endothelial cell proliferation (PH3, Fig. 3a, c) and apoptosis (TUNEL assay, Fig. 3b, d) in cavernous tissues, MCECs (Supplementary Fig. 6), and neuronal cells (MSCs and N2a, Supplementary Fig. 7) treated with MT-100 under diabetic or HG conditions. We found severely reduced proliferation and increased apoptosis under diabetic conditions both in vivo and in vitro. However, these effects were restored to normal levels after MT-100 treatment (Fig. 3, Supplementary Figs 6 and 7). The same effect was demonstrated in HUVECs exposed to HG (Fig. 3e–h). Collectively, these data suggest that MT-100 can restore vascular and neuronal cell contents by increasing proliferation and reducing apoptosis under diabetic conditions.

### MT-100 reduces permeability by increasing endothelial cell-to-cell junction protein levels and reducing reactive oxygen species production

**Fig. 4:**
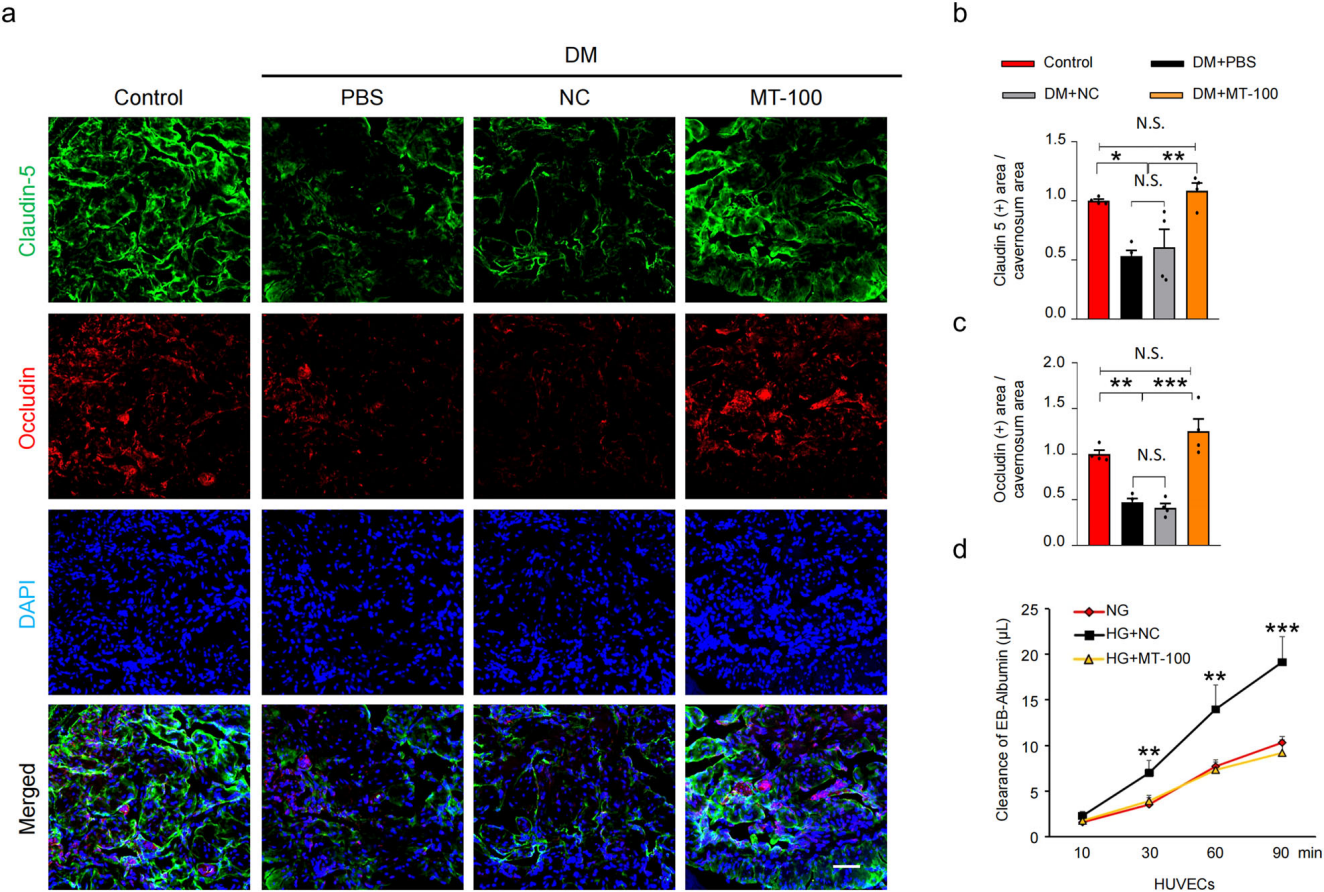
MT-100 reduces permeability under diabetic conditions. **a** Claudin-5 (green) and occludin (red) immunofluorescence staining of corpus cavernosum tissues from age-matched control and STZ-induced diabetic (DM) mice 2 weeks after repeated intracavernous injection (days −3 and 0) of phosphate-buffered saline (PBS, 20 μL), negative control IgG1 (NC, 10 μg in 20 μL of PBS), or MT-100 (10 μg in 20 μL of PBS). Nuclei were stained with DAPI (blue). Scale bars, 100 µm. **b**, **c** Quantitative analysis of claudin-5- and occludin-immunopositive areas using ImageJ software. The data are presented as mean ± SEM (*n* = 4). The relative ratio in the control groups was arbitrarily set to 1. *P < 0.05; **P < 0.01; ***P < 0.001. **d** The permeability assay was performed by measuring the leakiness of Evans blue (EB)-albumin in HUVECs treated with NC (10 μg in 20 μL of PBS) or MT-100 (10 μg in 20 μL of PBS) under normal glucose (NG) or high glucose (HG) conditions for 3 days. Clearance of EB-albumin was measured every 30 min up to 90 min. Data are presented as mean ± SEM (*n* = 4). **P < 0.01; ***P < 0.001. DAPI 4,6-diamidino-2-phenylindole, HUVECs human umbilical vein endothelial cells, N.S. not significant.

**Fig. 5:**
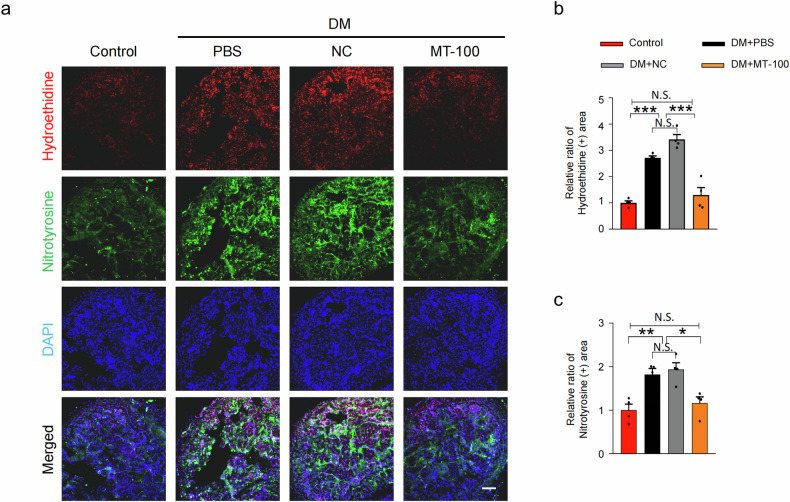
MT-100 decreases cavernous ROS production in diabetic mice. **a** In situ detection of superoxide anion (hydroethidine, red) and nitrotyrosine production (green) in corpus cavernosum tissues from age-matched control and STZ-induced diabetic (DM) mice 2 weeks after repeated intracavernous injection (days −3 and 0) of phosphate-buffered saline (PBS, 20 μL), negative control IgG1 (NC, 10 μg in 20 μL of PBS), or MT-100 (10 μg in 20 μL of PBS). Nuclei were stained with DAPI (blue). Scale bars, 100 µm. **b**, **c** Quantitative analysis of the ethidium bromide fluorescence-immunopositive cavernosum area (**b**) and nitrotyrosine-immunopositive cavernosum area (**c**) using ImageJ software. The data are presented as mean ± SEM (*n* = 4). The relative ratio in the control groups was arbitrarily set to 1. *P < 0.05; **P < 0.01; ***P < 0.001. DAPI 4,6-diamidino-2-phenylindole, ROS reactive oxygen species, N.S. not significant.

Endothelial junctions provide integrity to blood and lymph vessels and are essential for the formation of a vascular system^41^. DM reduces vascular integrity and increases extravasation, inducing vascular inflammatory responses and endothelial cell apoptosis in diabetic mice^42^. Therefore, to evaluate whether MT-100 also has a regulatory effect on the vascular permeability of corpus cavernosum tissue, we performed immunofluorescence staining for claudin-5 and occludin in diabetic mice. The expression of claudin-5 and occludin in the PBS- and NC-treated diabetic mice was lower than that in the nondiabetic controls, but MT-100 treatment significantly restored the levels of these endothelial cell-to-cell junction proteins in the cavernosum tissues of the diabetic mice (Fig. 4a–c). The permeability of HUVECs was then determined by measuring the leakage of Evans blue bound to bovine serum albumin under HG conditions. Leakage of Evans blue was significantly greater in HUVECs treated with NC under HG conditions. However, after treatment with MT-100, the leakage returned to normal levels (Fig. 4d). In addition, reactive oxygen species (ROS) production is associated with disturbances in the endothelial barrier and microvascular alterations under different pathological conditions^43^. Therefore, we also investigated the effect of MT-100 on ROS production. We used immunofluorescence staining of hydroethidine and nitrotyrosine to measure the levels of superoxide anion and peroxynitrite in the cavernous tissues of diabetic mice. The expression of hydroethidine and nitrotyrosine was significantly increased in the cavernosum tissues of diabetic mice but significantly decreased after treatment with MT-100 (Fig. 5). These results suggest that MT-100 can induce cavernous vessel integrity by increasing endothelial cell-to-cell junction protein levels and reducing cavernous ROS production in diabetic mice.

### Srpx2 is a novel target through which MT-100 restores erectile function in diabetic mice

**Fig. 6:**
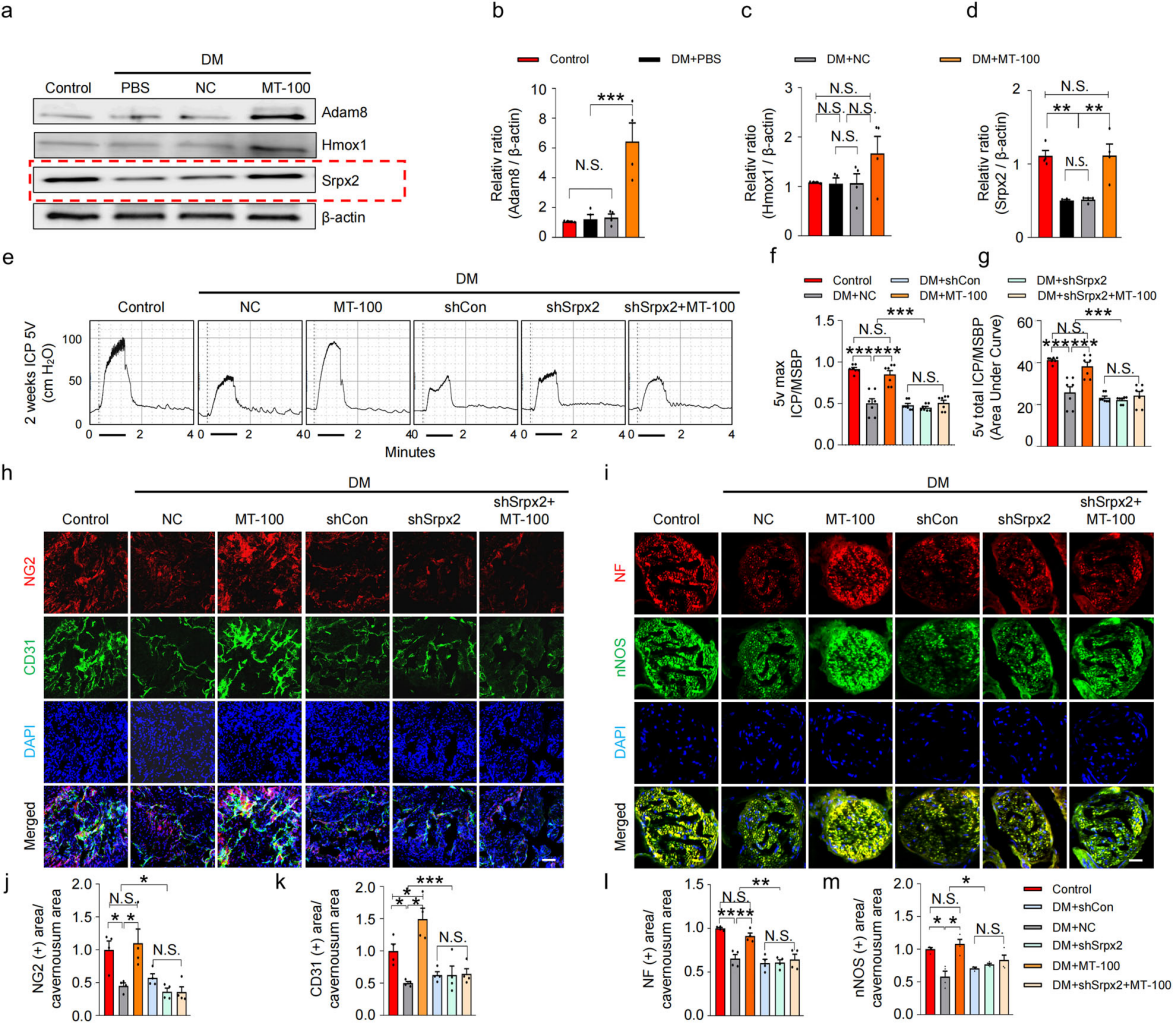
MT-100 improves erectile function via a novel target, Srpx2, in diabetic mice. **a** Representative Western blot for Adam8, Hmox1, and Srpx2 in corpus cavernosum tissues from age-matched control and STZ-induced diabetic (DM) mice 2 weeks after repeated intracavernous injection (days −3 and 0) of phosphate-buffered saline (PBS, 20 μL), negative control IgG1 (NC, 10 μg in 20 μL of PBS), or MT-100 (10 μg in 20 μL of PBS). **b**‒**d** Band intensity values for each neurotropic factor normalized to the density of β-actin and quantified using ImageJ software. The data are presented as mean ± SEM (*n* = 4). The relative ratio in the control group was defined as 1. **e** Representative intracavernous pressure (ICP) responses of age-matched control and DM mice stimulated after 2 weeks under the indicated conditions. The cavernous nerve was stimulated at 5 V. The stimulus interval is indicated by a solid bar. **f**, **g** Ratios of the mean maximal ICP (**f**) and total ICP (**g**, area under the curve) to the mean systolic blood pressure were calculated for each group (*n* = 7). **h**, **i** Representative images of immunofluorescence staining of corpus cavernosum (**h**) and dorsal nerve bundle (**i**) tissues for NG2 (red), CD31 (green), NF (red), and nNOS (green) after ICP studies. Nuclei were stained with DAPI (blue). Scale bars, 100 µm (**h**) and 25 µm (**i**). **j**‒**m** Quantitative analysis of corpus cavernosum pericyte (NG2), endothelial cell (CD31), and dorsal nerve bundle neuronal cell (NF and nNOS) content using ImageJ software. The data are presented as mean ± SEM (*n* = 4). The relative ratio in the control or normal glucose groups was arbitrarily set to 1. *P < 0.05; **P < 0.01; ***P < 0.001. DAPI 4,6-diamidino-2-phenylindole, N.S. not significant.

**Fig. 7:**
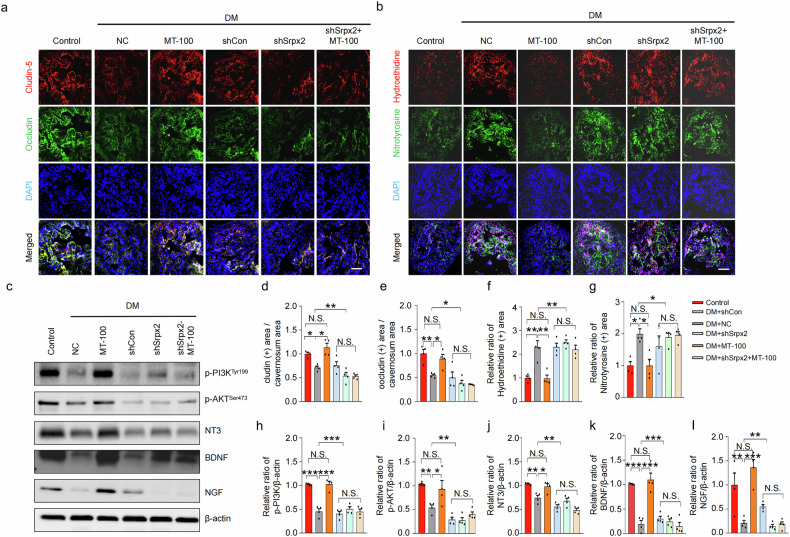
Identification of the mechanism by which MT-100/Srpx2 restores erections in diabetic mice. **a**, **b** Representative images of immunofluorescence staining of tight junction proteins (**a**, claudin-5 and occludin) and ROS production (**b**, hydroethidine and nitrotyrosine) under the indicated conditions. **c** Representative Western blot for p-PI3K^Tyr199^, p-AKT^Ser473^, NT3, BDNF, and NGF in corpus cavernosum tissues from the indicated groups. **d**–**l** Quantitative analysis of tight junction proteins (**d**, **e**), ROS production (**f**, **g**), and Western blots (**h**–**l**) using ImageJ software. The data are presented as mean ± SEM (*n* = 4). The relative ratio in the control or normal glucose groups was arbitrarily set to 1. *P < 0.05; **P < 0.01; ***P < 0.001. DAPI 4,6-diamidino-2-phenylindole, BDNF brain-derived neurotrophic factor, NGF nerve growth factor, NT-3 neurotrophin-3, DM STZ-induced diabetes, N.S. not significant.

To elucidate the specific target of MT-100 in the activation of the Tie2 signaling pathway, we performed RNA sequencing analyses of cavernosum tissues under various conditions. A total of 17,411 genes were detected in the four libraries, and 3153 significantly differentially expressed genes (DEGs) were selected on the basis of three conditions: fold change > 1.5, log2 > 4, and raw data expression >100. These genes were classified into 14 GO categories (Supplementary Fig. 8a, b) using ExDEGA software (E-Biogen, Inc., Korea). In a Venn diagram analysis, a total of 900 contra-regulated genes were detected between the DM + NC/Control and DM + MT-100/DM + NC groups (Supplementary Fig. 8c). Using the gene category selection tool, we selected genes related to angiogenesis (74 genes) from the contra-regulated genes and conducted further analysis and validation. Here, we list only the top 14 genes whose expression changed more than 1.5-fold in the DM + MT-100 vs. DM + NC comparison (Supplementary Fig. 8d). After performing Western blot verification of the top three genes (*Adam8, Hmox1*, and *Srpx2*), we found that the protein expression of Srpx2 was consistent with the results of the RNA sequencing analysis (Fig. 6a–d). After reviewing the literature on Srpx2, we hypothesized that Srpx2 may be involved in the ability of MT-100 to restore erectile function in diabetic mice. To evaluate whether Srpx2 is responsible for MT-100-induced neurovascular regeneration under diabetic conditions, we knocked down Srpx2 expression in mouse corpus cavernosum tissue via infection with different doses of lentivirus expressing small hairpin RNA (shRNA) targeting Srpx2 (shSrpx2, 5 × 10^3^, 2.5 × 10^4^, and 5 × 10^4^ TU/mL) or scrambled shRNA control (shCon) lentivirus (Supplementary Fig. 9). Erectile function was then assessed after the injection of MT-100. The maximal and total ICP relative to MSBP were significantly improved in diabetic mice treated with MT-100. However, these erectile function-improving effects of MT-100 were completely lost in the shSrpx2-infected group (Fig. 6e–h). Consistent with this observation, immunofluorescence staining of NG2, CD31, NF, and nNOS in corpus cavernosum tissue revealed that MT-100 significantly improved pericyte (Fig. 6h, j), endothelial cell (Fig. 6h, k), and neuronal cell (Fig. 6i, l, m) contents, which may be achieved by inducing proliferation and reducing cell apoptosis (as shown in Fig. 3) in diabetic mice. These effects were abolished by Srpx2 knockdown (Fig. 6h–m). Furthermore, in diabetic mice, the MT-100-mediated increase in endothelial cell-to-cell junction protein levels (Fig. 7a, d, e) and reduction in cavernous and mitochondrial ROS production (Fig. 7b, f, g and Supplementary Fig. 10) also occurred in a Srpx2-dependent manner (Fig. 7).

Next, to evaluate whether Srpx2 plays a direct role in inducing neurovascular regeneration, we treated MCECs and MPG tissues with Srpx2 directly to test whether Srpx2 itself affects neurovascular regeneration. First, we tested the concentration of Srpx2 involved in angiogenesis and found that treatment with 100 ng of Srpx2 could significantly induce tube formation under HG conditions, and this concentration was used in subsequent experiments (Supplementary Fig. 11a, b). We subsequently performed tube formation and MPG neurite sprouting experiments with Srpx2 or MT-100 treatment after infection with shSrpx2 lentivirus under HG conditions. We found that Srpx2 could significantly induce angiogenesis and MPG neurite sprouting, but after Sprx2 knockdown, the effect of MT-100 on neurovascular regeneration was significantly reduced (Supplementary Fig. 11c–f). In addition, we also found that the permeability of MCECs and MCPs was reduced after treatment with Srpx2, but these effects were abolished under Tie2 receptor knockdown conditions (Supplementary Fig. 11g–j). Finally, to explore this mechanism, we assessed the expression of p-PI3K^Tyr199^, p-AKT^Ser473^, NT3, BDNF, and NGF by Western blotting and eNOS^Ser1177^ phosphorylation by immunofluorescence staining (Supplementary Fig. 12). We found that the expression levels of these genes were significantly reduced in diabetic mice and that the expression of these targets was significantly restored after MT-100 treatment. This process was also accomplished in a Srpx2-dependent manner (Fig. 7c, h–l). Collectively, these results indicate that Srpx2 is a novel target of MT-100-regulated neurovascular regeneration in diabetic mice.

## Discussion

**Fig. 8:**
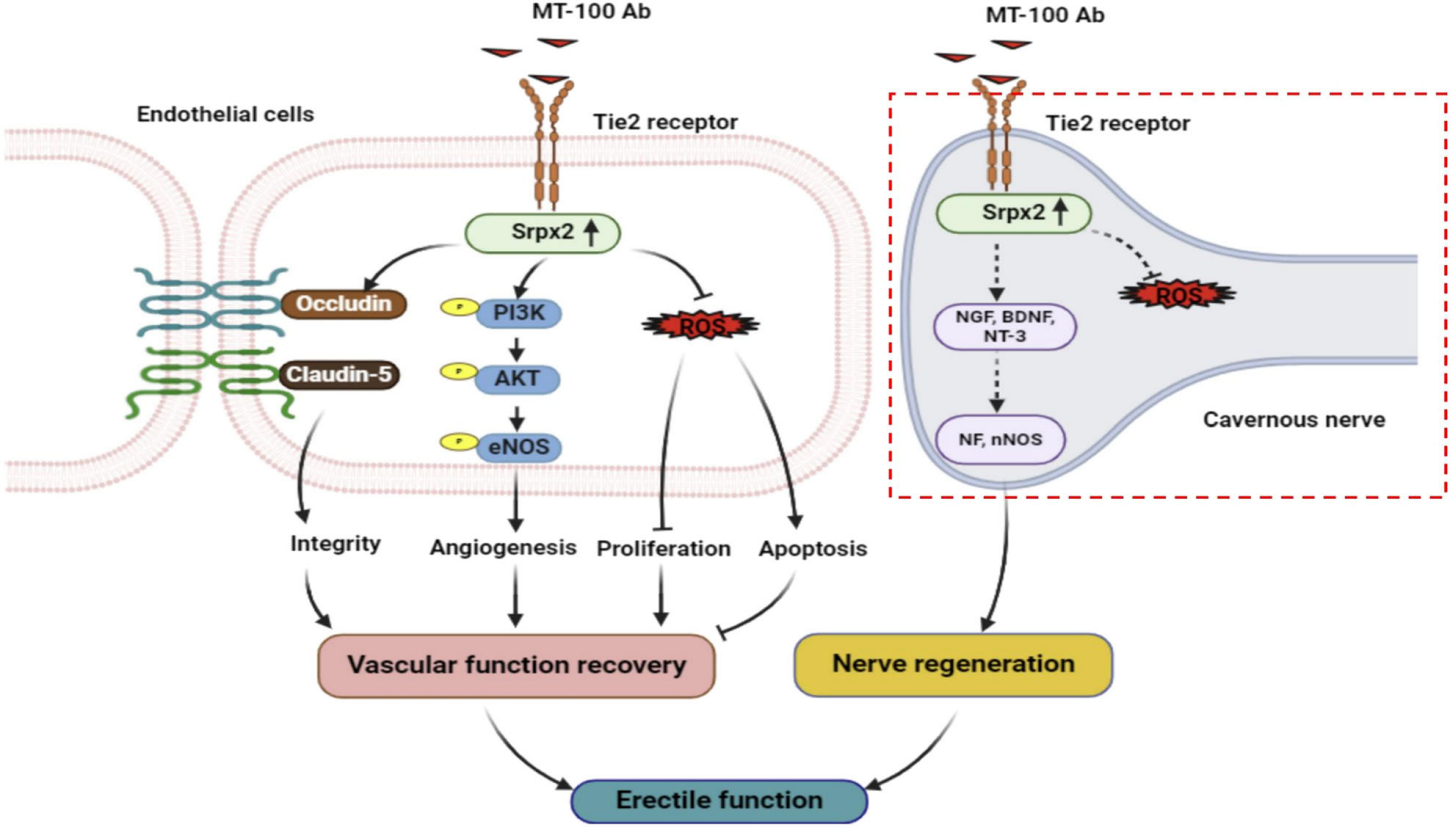
Schematic of the proposed mechanism by which MT-100 improved erectile function in diabetic mice.

ED related to DM occurs because persistently high blood sugar levels can severely damage nerves and vascular structures^35,44^. Although some treatments for ED with proteins and neurotrophic factors have had some success in the experimental phase^45,46^, their success in clinical trials has been limited due to their poor developability. In this study, we found that the human Tie2-agonistic antibody MT-100 can effectively induce angiogenesis and nerve regeneration through the novel target Srpx2, thereby improving erectile function in diabetic ED model mice. These conclusions are based on the following observations. First, MT-100 improves angiogenesis and neural regeneration, such as HUVEC and MCEC tube formation, aortic ring microvessel sprouting, and MPG neurite sprouting, thereby improving erectile function in diabetic mice. Second, through RNA sequencing analysis, we found that Srpx2 plays an important role in the process by which MT-100 promotes neurovascular regeneration. Through shSrpx2 lentivirus-mediated Srpx2 knockout experiments, we found that the effects of MT-100 were significantly attenuated under diabetic conditions. Figure 8 summarizes the proposed mechanism by which MT-100 alleviates diabetic ED.

Tie2 receptors and their angiopoietin ligands act as regulators of angiogenesis, cell survival, and vessel maturation^47,48^. Preclinical evidence suggests that modulating the Angpt/Tie2 pathway restores vascular stability and reduces inflammation^49^. In addition, the Angpt/Tie2 signaling pathway mediates neurite outgrowth in dorsal root ganglion neurons^50^. These studies indicate that drugs targeting the Tie2 signaling pathway constitute a novel therapeutic strategy for the clinical treatment of vascular or neurological diseases. A number of biopharmaceuticals targeting angpt (trebananib, nesvacumab, MEDI3617, vanucizumab, RG7716, REGN910-3, AKB-9778, and LY3127804) and Tie2 (regorafenib, rebastinib, altiratinib, ARRY-614, ABTAA, and hTAAB) are currently in preclinical or clinical development^16,51^. Tie2-targeted investigational drugs are in the early stages of development. One of the considerations in developing Tie2-agonistic antibodies is cross-reactive binding. Although there is approximately 93% amino acid sequence homology between human and mouse Tie2, several Tie2-agonistic antibodies reported thus far only bind to human Tie2^14,16^, which makes it difficult to evaluate the therapeutic efficacy of these antibodies in mouse disease models. In this context, MT-100 was rationally selected by screening with recombinant human and mouse Tie2; thus, its therapeutic activity could be verified using mouse ED models, as described in this study. In addition, because Ang-2 is overexpressed in the diabetic ED model and can inhibit the Tie2 signaling pathway^12^, Tie2-agonistic antibodies hinder the functional activity of excess Ang-2. MT-100 not only through direct Tie2 activation but also by interfering with Ang-2 binding to Tie2 (Fig. 1e, g). Therefore, we suggest that MT-100 has dual functions: activating Tie2 and blocking the binding of Ang-2 to Tie2. In this study, MT-100 had angiogenic effects on mouse cells despite being a human Tie2-agonistic antibody. We used a diabetes-induced ED mouse model to evaluate the angiogenic effects of MT-100 and its optimal therapeutic dose in vivo. This model has been utilized in many studies on microvascular complications caused by type 1 DM^7,52^. Consistently, studies have shown that exogenous injection of MT-100 into the penis of diabetic mice can significantly improve the survival of cavernous vascular and neuronal cells and increase blood vessel stability in the corpus cavernosum, thereby improving erectile function in diabetic mice. However, we could not directly isolate penile neuronal cells for in vitro experiments. We could perform only indirect immunofluorescence staining of the dorsal nerve bundle of penis tissue and harvest the MPG for ex vivo neurite sprouting experiments. It may be interesting to examine the neuroregenerative effects of MT-100 in other animal models of neurological injury, such as cavernous nerve injury models^30^, sciatic nerve injury models^53,54^, and spinal cord injury models^55^. These findings suggest that local injection of MT-100 may be a promising strategy for the treatment of diabetic ED.

Diabetic complications are known to increase ROS and oxidative stress, leading to cell death through multiple mechanisms and, ultimately, tissue damage^56^. For example, high levels of ROS induce cell death, apoptosis, and senescence. In contrast, low levels of ROS act as signaling molecules that mediate cell growth, migration, differentiation, and gene expression^57^. In addition, previous studies have shown that Ang-1 induces cytoplasmic ROS production in a rapid but transient Tie-2-dependent manner, and the molecular sources of these ROS have never been clearly identified^58^. However, we found that MT-100 can activate the Tie2 signaling pathway and reduce the expression of superoxide anion (hydroethidium) and peroxynitrite anion (nitrotyrosine) in diabetic mice. These findings suggest that the process by which MT-100 regulates the ROS balance may not involve a Tie2-dependent signaling pathway. There may be other unknown pathways that require further experimental investigation.

To determine the potential mechanism of action of MT-100 in promoting erectile function in diabetic ED mice, we performed RNA sequencing analysis and identified Srpx2 as a novel target of MT-100. Srpx2 is associated with synaptic plasticity, tissue remodeling, and angiogenesis^59^. Srpx2 may play an important role in vascular remodeling and the recovery of synaptic loss caused by spinal cord injury^60^. Silencing Srpx2 via small interfering RNA (siRNA) causes mesenchymal endothelial cell migration and delays proliferation and budding^61^. In this study, we found that Srpx2 expression was significantly increased in diabetic mice treated with MT-100. Given that Srpx2 expression increased after MT-100 treatment, we hypothesized that MT-100 may indirectly induce an increase in Srpx2 expression by regulating several factors, which is a complex process. However, we cannot prove and explain this in the current study, and further experiments are needed to demonstrate how MT-100 increases Srpx2 expression under diabetic conditions. In addition, the neurovascular regeneration effect induced by MT-100 in diabetic mice was significantly abolished after Srpx2 knockout. Previous studies have shown that the Tie2 signaling pathway not only induces endothelial cell survival but also promotes neural development and neurite outgrowth through the PI3K and Akt kinase signaling pathways^15,62^. Therefore, when we explored the mechanism of action of MT-100, we also observed the phosphorylation of eNOS, PI3K, and AKT, as well as the expression of neurotrophic factors (NGF, BDNF, and NT3). These results indicate that MT-100 can simultaneously activate the Tie2 and Srpx2 signaling pathways. However, we were unable to demonstrate the relationship between Tie2 and Srpx2, but this finding at least indicates that the process by which MT-100 regulates the ROS balance may be achieved through the Srpx2 signaling pathway, not the Tie2 signaling pathway. This requires further investigation.

Taken together, our results demonstrate the efficacy of MT-100 in inducing neurovascular regeneration in diabetic patients. As a new target, Srpx2 plays an important role in this process. As a human Tie2-agonistic antibody, MT-100 may more readily enter clinical evaluation for the treatment of diabetic ED. This work not only expands our understanding of diabetic ED but also has important clinical implications for the treatment of diabetic ED and other DM-related complications.

## Supplementary information

Supplementary Information

## Data Availability

All study data are included in the article and/or Supplemental Information. All other data and materials used for the analysis are available from the corresponding author upon reasonable request.
