# Supplementary material for: MT-100, a human Tie2-agonistic antibody, improves penile neurovasculature in diabetic mice via the novel target Srpx2
Source: Exp Mol Med. 2025 Jan 1;57(1):104–17. doi: 10.1038/s12276-024-01373-1 (PMC11799434; doi:10.1038/s12276-024-01373-1)
Supplement: Supplementary file 1 — Supplementary Information [file 12276_2024_1373_MOESM1_ESM.pdf]

Supplementary Information

MT-100, a human Tie2-agonistic antibody, improves penile neurovasculature in diabetic mice via the novel target Srpx2

Fang-Yuan Liu[a,1], Young-Lai Cho[b,1], Fitri Rahma Fridayana[a,c], Lashkari Niloofar[a,c], Minh Nhat Vo[a], Yan Huang[a,c], Limanjaya Anita[a], Mi-Hye Kwon[a], Jiyeon Ock[a], Seon-Jin Lee[b,d], Guo Nan Yin[a,*], Nam-Kyung Lee[e,f,*], and Ji-Kan Ryu[a,c,*]

*Corresponding Authors

Ji-Kan Ryu, Email: rjk0929@inha.ac.kr; Nam-Kyung Lee, Email: nklee@kribb.re.kr; Guo Nan Yin, Email: yinguonan320@naver.com

This PDF file includes:

Supplementary Fig. 1 to 12
Supplementary Table 1 to 2

Supplementary Figures

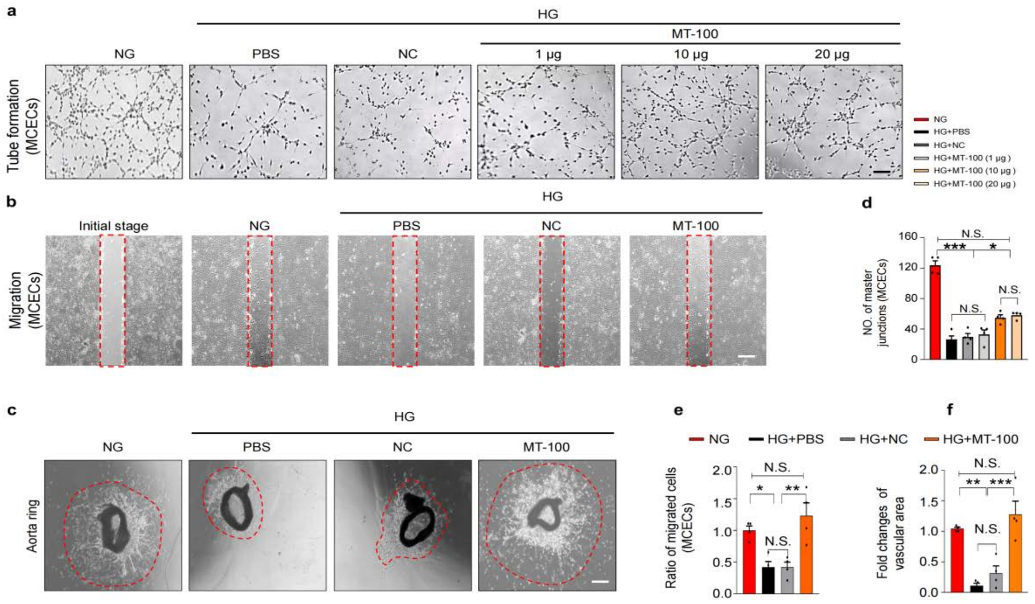

Supplementary Fig. 1 MT-100 promotes angiogenesis under high-glucose conditions.

a Tube formation assay. MCECs were treated with phosphate-buffered saline (PBS), negative control IgG1 (NC, 10 μg/mL) and MT-100 (1μg, 10 μg, 20 μg/mL, respectively) under normal-glucose (NG) or high-glucose (HG) for 3 days. Scale bars, 100 μm. **b** Migration assay in MCECs 24 hours after treatment with PBS, negative control IgG1 (NC, 10 μg/mL) and MT-100 (10 μg/mL) under NG or HG for 3 days. Scale bars, 100 μm. **c** Aortic Ring Assay. Aorta ring were treated with PBS, negative control IgG1 (NC, 10 μg/mL) and MT-100 (10 μg/mL) under NG or HG for 7 days. Scale bars, 100 μm. **d** Quantification of number of master junctions per field (n = 4). **e** The ratio of migrated MCECs in the frame line was quantified (n = 4). **f** Intensity of microvessel sprouting area from aortic rings (n = 4). Quantitative analysis was performed by using ImageJ software. The results were presented as means ± SEM.

The relative ratio in the NG groups was arbitrarily set to 1. *P < 0.05; **P < 0.01; ***P < 0.001. MCECs: mouse cavernous endothelial cells.

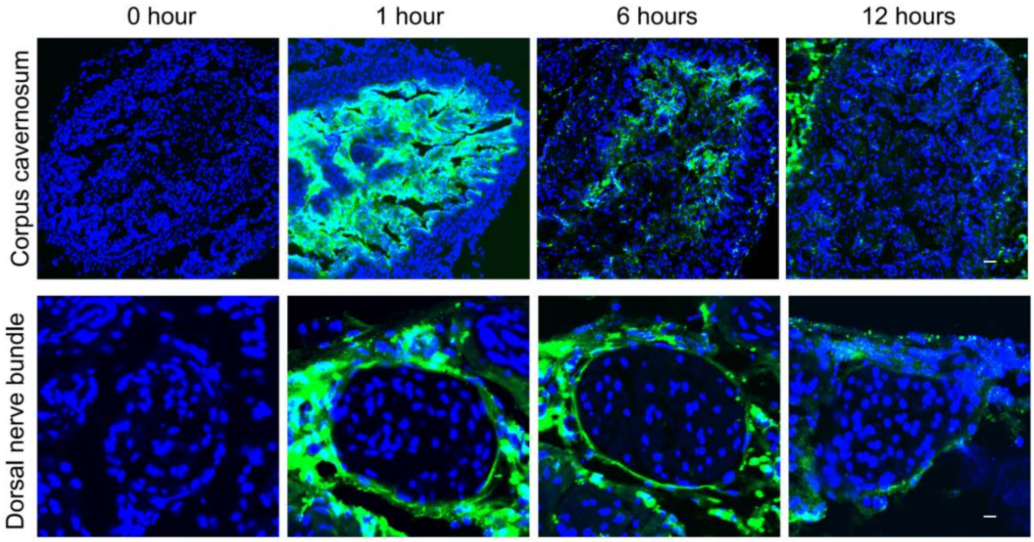

Supplementary Fig. 2 In vivo detection of MT-100 in the penis tissue of mice.

The penis tissue was harvested 0, 1, 6, and 12 hours after intracavernous injection of FITC-conjugated MT-100 (10 μg/20 μL) into the normal mice. Nuclei were stained by DAPI (blue). Scale bars, 100 μm (corpus cavernosum) and 25 μm(dorsal nerve bundle). DAPI, 4,6-diamidino-2-phenylindole.

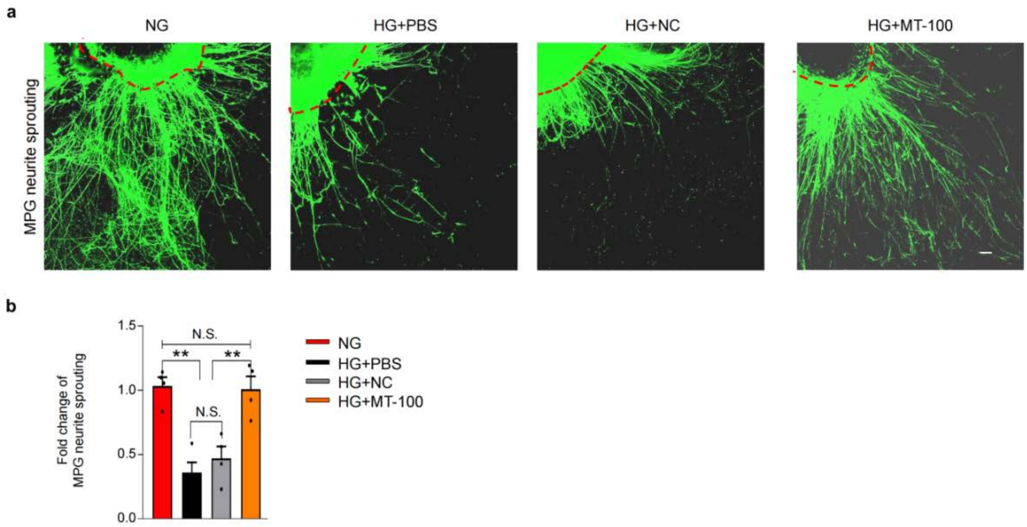

Supplementary Fig. 3 MT-100 induces major pelvic ganglion (MPG) neurite

outgrowth under high-glucose conditions.

a Neurofilament (NF, green) immunofluorescent staining in mouse MPG tissues after treatment with PBS, negative control IgG1 (NC, 10 μg/mL) and MT-100 (10 μg/mL) under normal-glucose (NG) or high-glucose (HG) for 5 days. Scale bars, 100 μm. **b** Quantitative analysis of NF-immunopositive neurite length from MPG tissues by using ImageJ software. Results are presented as means ± SEM (n = 4). The relative ratio in the NG groups was arbitrarily set to 1. **P < 0.01. N.S., not significant.

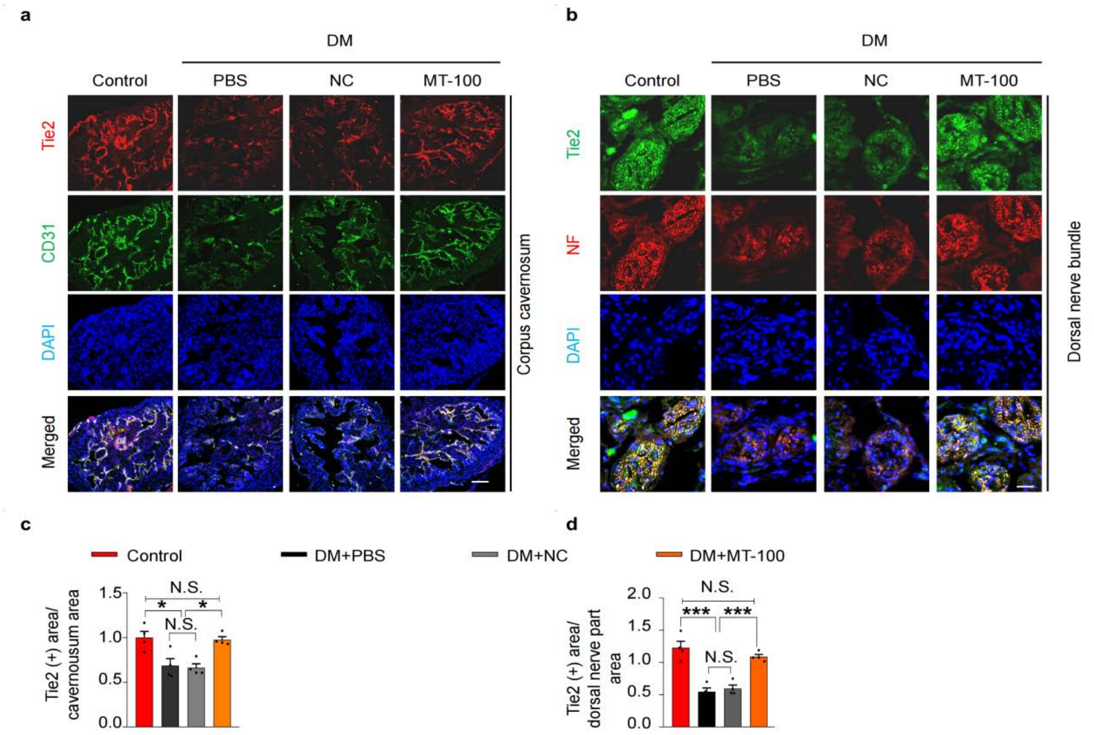

Supplementary Fig. 4 MT-100 induces Tie2 expression in diabetic mice.

a and b Immunofluorescent staining for Tie2/CD31 in corpus cavernosum (**a**) and Tie2/NF in dorsal nerve bundle (**b**) tissues from age-matched control and DM mice 2 weeks after repeated intracavernous injection (days −3 and 0) of PBS (20 μL), NC (10 μg in 20 μL PBS), and MT-100 (10 μg in 20 μL PBS) after ICP studies. Nuclei were stained by DAPI (blue). Scale bars, 100 μm (**a**, corpus cavernosum) and 25 μm(**b**,

dorsal nerve bundle). **c and d** Quantitative analysis of Tie2 expression in endothelial cells (**c**) and neuronal cells (**d**) by using ImageJ software. Data are presented as mean ± SEM (n = 4). The relative ratio in the control groups was arbitrarily set to 1. *P < 0.05; ***P < 0.001. DAPI, 4,6-diamidino-2-phenylindole; NF, Neurofilament; N.S., not significant.

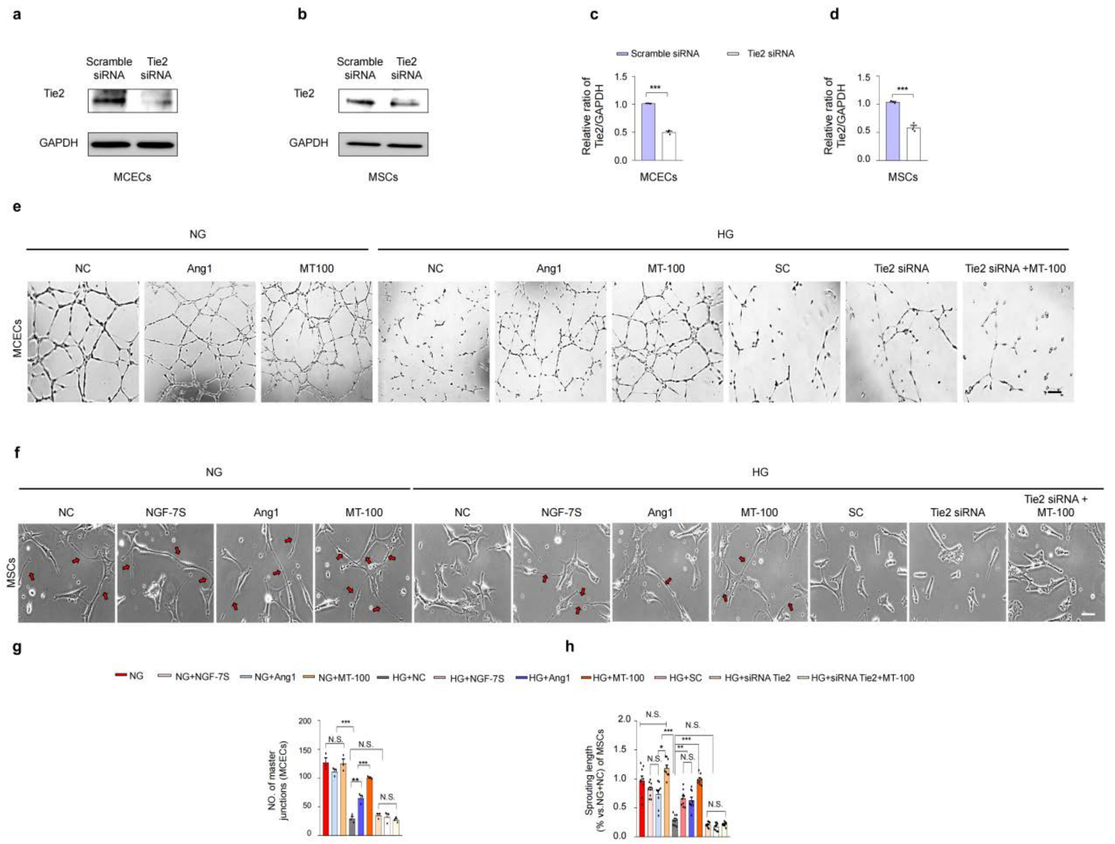

Supplementary Fig. 5 MT-100 induces tube formation and mouse schwann cells (MSCs) neurite sprouting via the Tie2 receptor under high-glucose conditions.

a and b Representative western blots for Tie2 in MCECs (**a**) and MSCs (**b**) transfected with scramble siRNA or Tie2 siRNA. **c and d** Band intensity values for Tie2 normalized to the density of GAPDH in MCECs (**c**) and MSCs (**d**) quantified by using Image J software. Data are presented as mean ± SEM (n = 3). The relative ratios

of the scramble siRNA group were set to 1. ***P<0.001. **e** Tube formation assay. One day after transfected with scramble siRNA or Tie2 siRNA, the MCECs were treated with negative control IgG1(NC, 10 μg/mL), Ang1 (200 ng/mL), MT-100 (10 μg/mL) under normal glucose (NG, 5 mM) or high glucose (HG, 30 mM) condition for other 3 days. Scale bars, 100 μm. **f** MSCs neurite sprouting assay. One day after transfected with scramble siRNA or Tie2 siRNA, the MSCs were treated with negative control IgG1(NC), nerve growth factor-7S (NGF-7S, 10 ng/mL), Ang1 (200 ng/mL), MT-100 (10 μg/mL) under normal glucose (NG, 5 mM) or high glucose (HG, 30 mM) condition for other 3 days. Scale bars, 50 μm. **g and h** Quantification of the number of master junctions per field (**g**, n = 3) and neurite length (**h**, n = 9) were quantified by using Image J software. Data are presented as mean ± SEM. The relative ratios of the negative control group were set to 1 (**h**). *P<0.05; **P<0.01; ***P<0.001. MCECs, mouse cavernous endothelial cells; N.S., not significant.

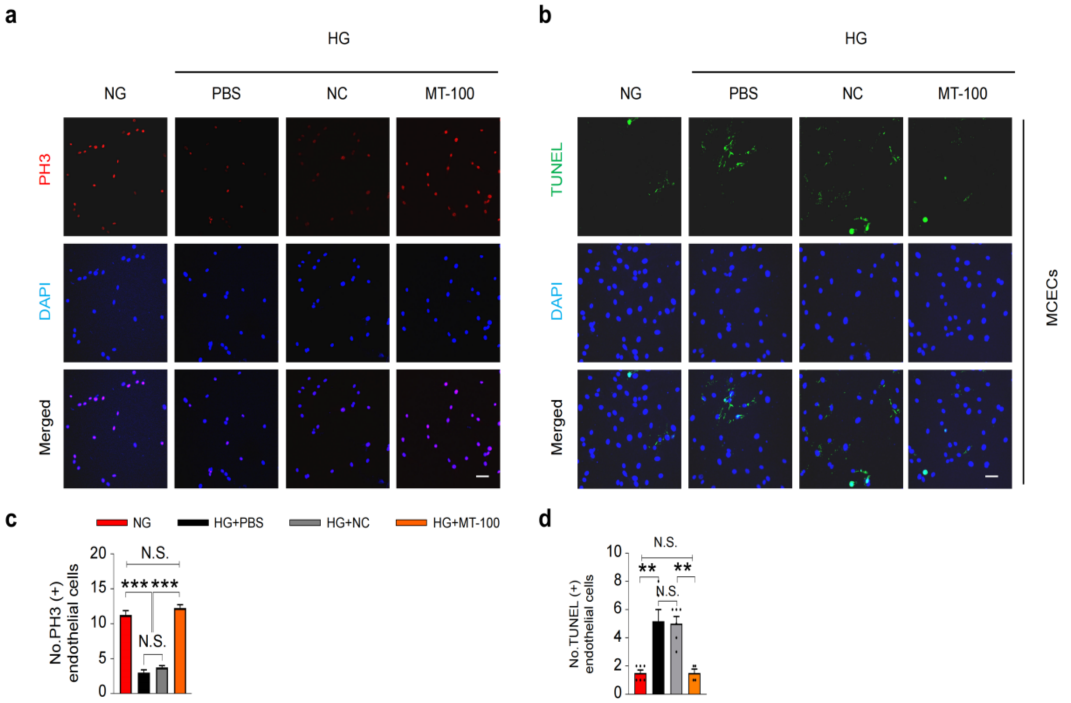

Supplementary Fig. 6 MT-100 increases proliferation and decreases apoptosis of MCECs under high-glucose conditions.

a and b *In vitro* studies of proliferation assay (PH3; **a**) and TUNEL assay (**b**) in MCECs, treated with PBS, negative control IgG1 (NC, 10 μg/mL) and MT-100 (10 μg/mL) under normal-glucose (NG) or high-glucose (HG) for 3 days. Nuclei were stained by DAPI (blue). Scale bars, 25 μm. **c and d** Number of PH3-positive (**c**) and TUNEL-positive (**d**) MCECs, quantified by using ImageJ software. Results are presented as means ± SEM (n = 4). **P < 0.01; ***P < 0.001. DAPI, 4,6-diamidino-2-phenylindole; TUNEL, terminal deoxynucleotidyl transferase-mediated deoxyuridine triphosphate nick end labeling; MCECs: mouse cavernous endothelial cells; N.S., not significant.

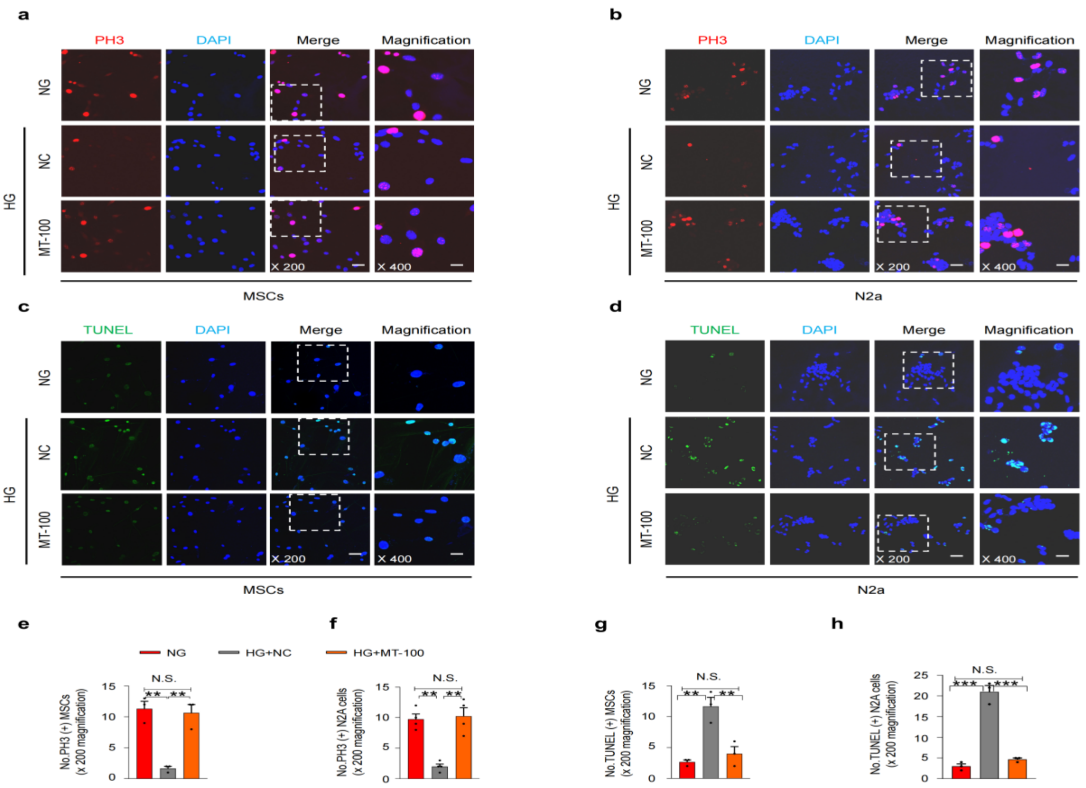

Supplementary Fig. 7 MT-100 increases proliferation and decreases apoptosis of

neuronal cells under high-glucose conditions.

a and b *In vitro* studies of proliferation assay (PH3, red) in MSCs (**a**) and N2a (**b**) treated with negative control IgG1 (NC, 10 μg/mL) and MT-100 (10 μg/mL) under normal-glucose (NG) or high-glucose (HG) conditions for at least 3 days. Nuclei were stained with DAPI (blue). Scale bars, 50 μm (merge image) 25 μm (magnification image). **c and d** *In vitro* studies of TUNEL (green) assay in MSCs (**c**) and N2a (**d**) treated with same conditions described above. **e-h** Number of PH3-positive and TUNEL-positive cells quantified by using ImageJ software. Results are presented as means ± SEM (n = 3). **p < 0.01; ***p<0.001. TUNEL, terminal deoxynucleotidyl transferase-mediated deoxyuridine triphosphate nick end labeling; DAPI, 4,6-diamidino-2-phenylindole; MSCs: mouse schwann cells. N2a: Neuro-2a; N.S.= not significant.

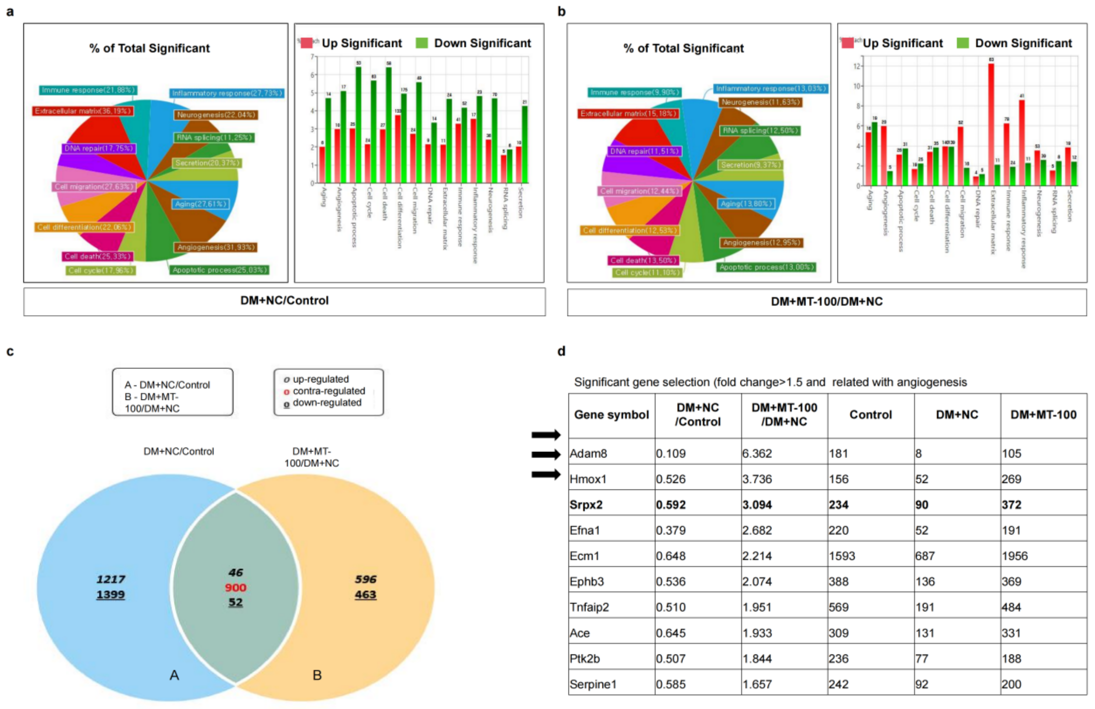

Gene symbol	DM+NC /Control	DM+MT-100 /DM+NC	Control	DM+NC	DM+MT-100
Adam8	0.109	6.362	181	8	105
Hmox1	0.526	3.736	156	52	269
**Srpx2**	**0.592**	**3.094**	**234**	**90**	**372**
Efna1	0.379	2.682	220	52	191
Ecm1	0.648	2.214	1593	687	1956
Ephb3	0.536	2.074	388	136	369
Tnfaip2	0.510	1.951	569	191	484
Ace	0.645	1.933	309	131	331
Ptk2b	0.507	1.844	236	77	188
Serpine1	0.585	1.657	242	92	200

Supplementary Fig. 8 Significant differentially expressed genes (DEGs) of the RNA-sequencing data were distributed to gene ontology (GO) categories.

a The distribution of total significantly DEGs in 14 GO categories in the DM + negative control IgG1 (NC, 10 μg in 20 μL PBS) group compared with the age-matched control group. **b** The distribution of total significantly DEGs in 14 GO categories in the DM +MT-100 (10 μg in 20 μL PBS) group compared with the DM + NC group. Pie chart represents total percentage; Bars graph represents detailed percentage and numbers of upregulated and downregulated genes, respectively. **c** Veen Diagram analysis between DM+NC/Control and DM+MT-100/DM+NC. **d** Top 10 contra-regulated genes showed increases or decreases in DM+NC/Control and DM+MT-100/DM+NC expression ratios grater than 1.5.

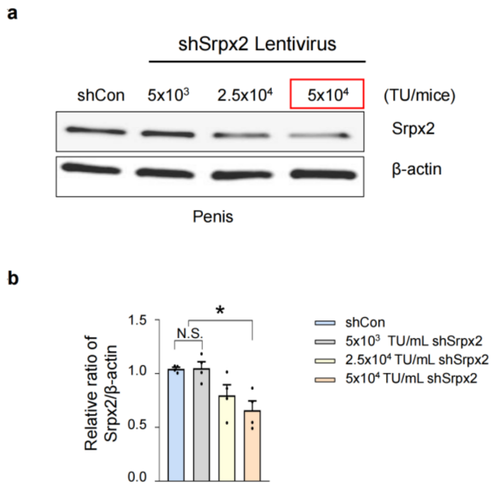

Supplementary Fig. 9 Knockdown of Srpx2 in penis tissues.

a Representative data for Srpx2 in mouse penis tissues infected with shCon or shSrpx2 lentivirus at three doses (5 x10^3, 2.5 x10^4, or 5 x10^4 TU/mice) for at least 3 days. **b** Quantitative analysis of Srpx2 protein expression using ImageJ software. Data

are presented as mean ± SEM (n = 4). The relative ratio in the shCon groups was arbitrarily set to 1. *P < 0.05. N.S., not significant.

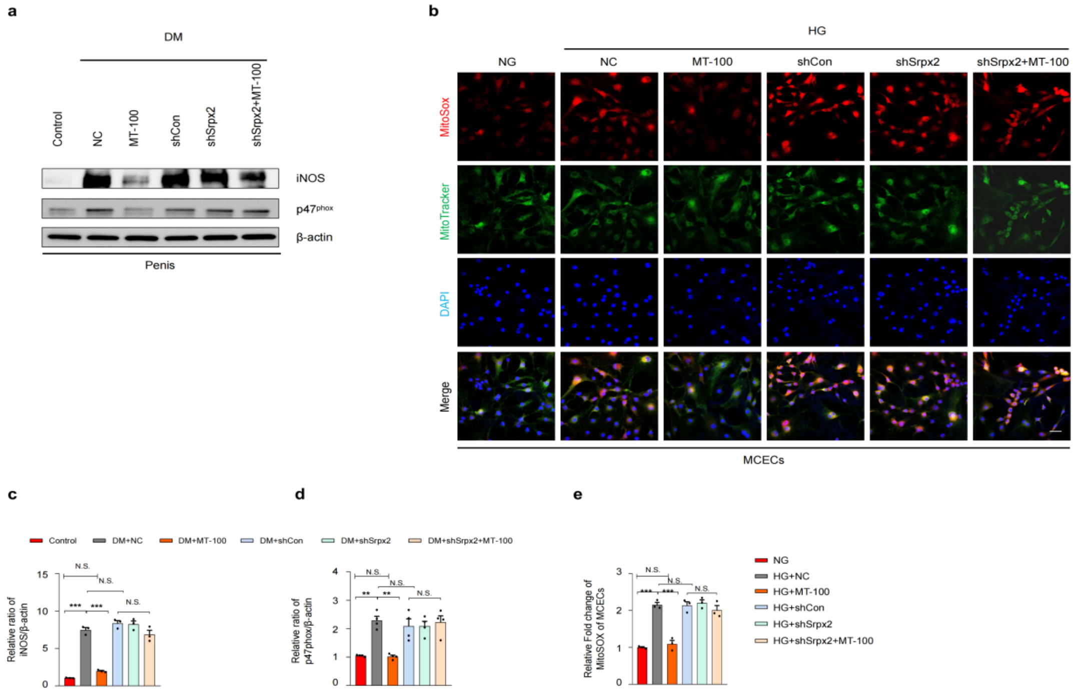

Supplementary Fig. 10 MT-100 decreases cavernous ROS production via a novel target, Srpx2, in diabetic mice.

a Respective western blot for iNOS, p47phox, MT-ND1 in corpus cavernosum tissues under the indicated conditions. **b** Mitochondria ROS detection. Immunofluorence staining with MitoSox red (for mitochondria superoxide) and MitoTracker (green) in MCECs under the indicated conditions. Scale bars, 50 µm. Nuclei were stained by DAPI (blue). **c and d** Band intensity values for iNOS (**c**), p47phox (**d**) normalized to the density of β-actin in corpus cavernosum tissues quantified by using Image J software. Data in graphs are presented as mean ± SEM (n =3). The relative ratio in the control group was arbitrarily set to 1. **e** Quantification of the relative ratio of MitoSox positive area by using Image J software. Data in graphs are presented as mean ± SEM

(n =3). The relative ratio in the NG groups was arbitrarily set to 1. **P < 0.01; ***P < 0.001. MCECs, mouse cavernous endothelial cells; DAPI, 4,6-diamidino-2-phenylindole; N.S. = not significant.

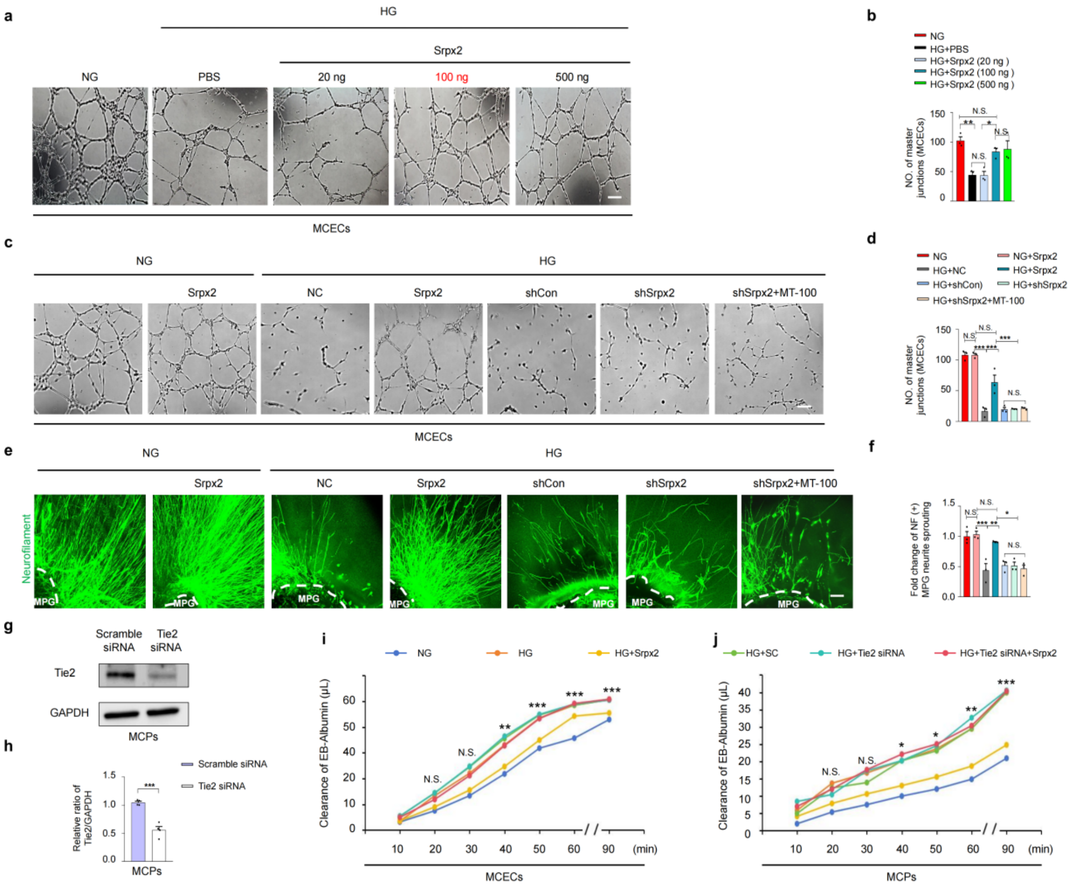

Supplementary Fig. 11 MT-100 induces angiogenesis and neurite sprouting via a novel target Srpx2 under high-glucose conditions.

a Tube formation assay. MCECs were treated with PBS and Srpx2 protein (20 ng, 100 ng, 500 ng/mL, respectively) under high-glucose (HG) conditions. Scale bars, 100 µm. **b** Quantification of the number of master junctions per field (n = 3). **c** Tube formation assay. MCECs were infected with shCon or shSrpx2 lentivirus (5 x10^4 TU/mL) for at least 3 days, then the cells were treated with negative control IgG1 (NC, 10 µg/mL), Srpx2 protein (100 ng/mL) and MT-100 (10 µg/mL) under normal-glucose (NG)

conditions or HG conditions, respectively. Scale bars, 100 µm. **d** Quantification of the number of master junctions per field (n = 3). **e** Neurofilament (NF, green) immunofluorescent staining in mouse MPG tissues with same conditions described above. Scale bars, 100 µm. **f** Fold change of NF-immunopositive neurite sprouting from MPG tissues were quantified using ImageJ software. Data in graphs are presented as mean ± SEM (n =3). The relative ratio in the NG groups was arbitrarily set to 1. **g** Representative western blots for Tie2 in MCPs treated with scramble siRNA or Tie2 siRNA. **h** Band intensity values for Tie2 normalized to the density of GAPDH in MCPs quantified by using Image J software. Data in graphs are presented as mean ± SEM (n =3). The relative ratio in the scramble siRNA group was arbitrarily set to 1. **i and j** Permeability assay. One day after transfected with scramble siRNA (SC) or Tie2 siRNA, the MCECs and MCPs were treated with Srpx2 protein (100 ng/mL) under NG or HG condition for other 3 days. The clearance of EB-Albumin in MCECs (**i**) and MCPs (**j**) during 90 min were detected. *P<0.05; **P < 0.01; ***P < 0.001. MCECs, mouse cavernous endothelial cells; N.S. = not significant.

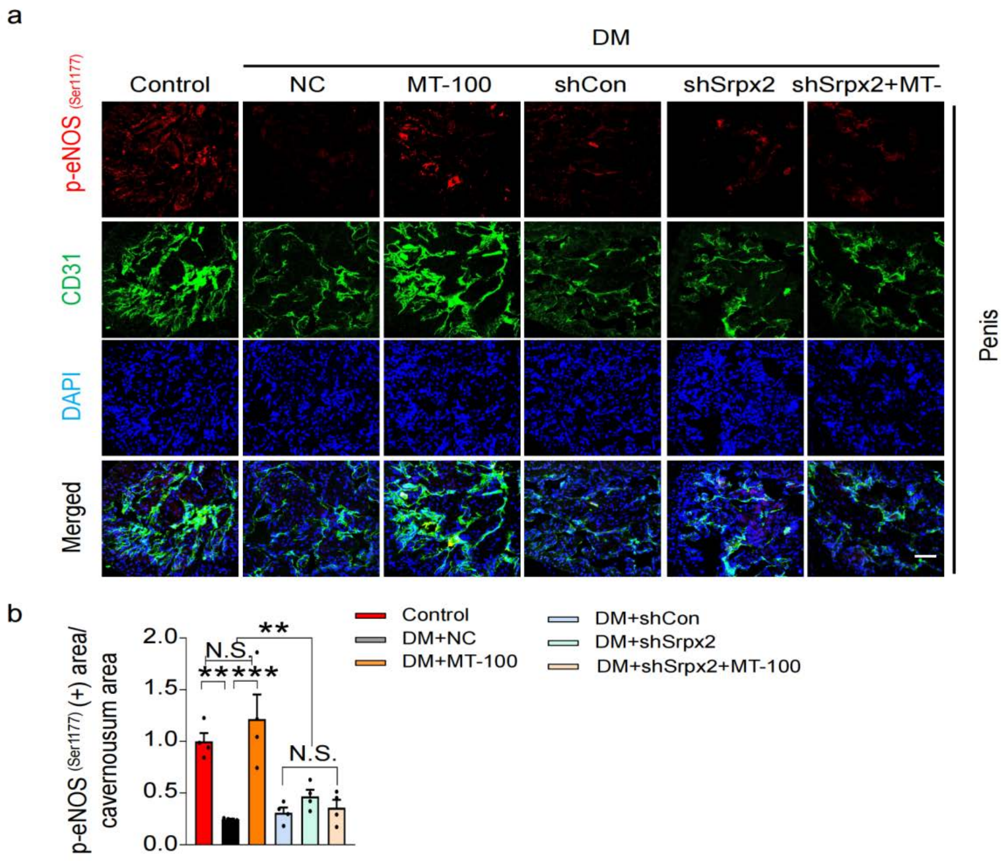

Supplementary Fig. 12 MT-100 induces cavernous eNOS phosphorylation via a novel target Srpx2 in diabetic mice.

a Representative images of immunofluorescent staining of corpus cavernosum tissues from indicated conditions for p-eNOS^{ser1177} (red) and CD31 (green) after ICP studies. Nuclei were stained by DAPI (blue). Scale bars, 100 µm. **b** Quantitative analysis of the p-eNOS^{ser1177}-immunopositive cavernosum area using ImageJ software. Data are presented as mean ± SEM (n = 4). The relative ratio in the control groups was arbitrarily set to 1. **P < 0.01; ***P < 0.001. DAPI, 4,6-diamidino-2-phenylindole; NC, negative control IgG1; DM, STZ-induced diabetic; shCon, scrambled control shRNA; shSrpx2, shRNA targeting Srpx2; N.S., not significant.

Supplementary Tables

Supplementary Table 1. Physiologic and metabolic parameters: 2 weeks after treatment with PBS, negative control (NC) or MT-100 (1 μg, 10 μg, 20 μg) in DM

	Control	DM-PBS	DM+NC	DM+ MT-100 (1 μg)	DM+ MT-100 (10 μg)	DM+ MT-100 (20 μg)
Body weight(g)	31.11±1.36	22.11±1.45*	23.17±1.77*	22.07±1.08*	22.83±1.87*	22.10±1.71*
Postprandial glucose(mg/dl)	135.29±14.6	523.86±66.7*	541±61.1*	530±82.53*	540.71±42.81*	523±59.81*
Fasting glucose(mg/dl)	122.43±12.04	366±48*	361.57±65.2*	332±54.63*	333.29±58.14*	338±63.58*
MSBP (mm Hg)	132.7±10.85	139.5±9.87	137.55±10.69	145.71±10.58	138.33±7.44	137.36±5.44

Values are the mean ± SEM for n = 7 animals per group. *P < 0.001; MSBP, mean systolic blood pressure.

Supplementary Table 2. Physiologic and metabolic parameters: 2 weeks after treatment with negative control (NC), MT-100, shCon, shSrpx2 or shSrpx2+MT-100

	Control	DM+NC	DM+MT-100	DM+shCon	DM+shSrpx2	DM+shSrpx2+MT-100
Body weight(g)	31.7±1.1	23.5±0.9*	23.3±2.1*	23.0±1.7*	22.6±1.7*	23.9±2*
Postprandial glucose(mg/dl)	146.9±22.3	543.4±40.1*	527±52.4*	524.9±63.9*	573.1±39*	528.1±65.2*
Fasting glucose(mg/dl)	82.6±11.4	265.6±76.3*	265.9±54.0*	257.6±66.3*	255.6±90.0*	263.86±91.0*
MSBP (mm Hg)	123.6±12.9	121.1±15.5	125±14.7	125±10.1	124.7±8.4	123.5±10.0

Values are the mean ± SEM for n = 7 animals per group. *P < 0.001; MSBP, mean systolic blood pressure.
